# Lipid droplet availability affects neural stem/progenitor cell metabolism and proliferation

**DOI:** 10.1038/s41467-021-27365-7

**Published:** 2021-12-21

**Authors:** Mergim Ramosaj, Sofia Madsen, Vanille Maillard, Valentina Scandella, Daniel Sudria-Lopez, Naoya Yuizumi, Ludovic Telley, Marlen Knobloch

**Affiliations:** 1 Department of Biomedical Sciences, University of Lausanne, Lausanne, Switzerland; 2 Graduate School of Pharmaceutical Sciences, The University of Tokyo, Tokyo, Japan; 3 Department of Fundamental Neurosciences, University of Lausanne, Lausanne, Switzerland

**Keywords:** Organelles, Neurogenesis, Fat metabolism, Neural stem cells

## Abstract

Neural stem/progenitor cells (NSPCs) generate new neurons throughout adulthood. However, the underlying regulatory processes are still not fully understood. Lipid metabolism plays an important role in regulating NSPC activity: build-up of lipids is crucial for NSPC proliferation, whereas break-down of lipids has been shown to regulate NSPC quiescence. Despite their central role for cellular lipid metabolism, the role of lipid droplets (LDs), the lipid storing organelles, in NSPCs remains underexplored. Here we show that LDs are highly abundant in adult mouse NSPCs, and that LD accumulation is significantly altered upon fate changes such as quiescence and differentiation. NSPC proliferation is influenced by the number of LDs, inhibition of LD build-up, breakdown or usage, and the asymmetric inheritance of LDs during mitosis. Furthermore, high LD-containing NSPCs have increased metabolic activity and capacity, but do not suffer from increased oxidative damage. Together, these data indicate an instructive role for LDs in driving NSPC behaviour.

## Abstract (web summary)

Lipid droplets (LDs) are central to lipid metabolism, which is known to regulate neural stem cell behavior. Here, the authors show that LDs influence neural stem cell proliferation and metabolic activity and change upon differentiation or quiescence.

## Introduction

The regulatory mechanisms that determine stem cell quiescence, self-renewal, proliferation, or differentiation have been the focus of intense research over the past decades^1^. Cellular metabolism has emerged as a key regulator of this balance in several stem cell types^2^. While stem cells are primarily in a glycolytic state, their differentiated progeny shift to oxidative metabolism. Manipulations of this balance have revealed that this metabolic pattern is not only a signature of a specific stem cell state, but also has functional consequences such as increased reprogramming capacity when glycolysis is enforced^3^ or prevented differentiation if oxidative metabolism is hindered^4,5^. The importance of other metabolic pathways for stem cell regulation is just emerging. Several recent studies have discovered that lipid metabolism is crucial for the regulation of different types of adult stem cells such as hematopoietic stem cells^6^, muscle satellite stem cells^7^, and intestinal stem cells^8,9^.

Lipid metabolism is also crucial for neural stem/progenitor cells (NSPCs). NSPCs persist throughout adulthood in most mammalian brains^10^. In rodents, NSPCs reside in at least two regions, the subventricular zone (SVZ) of the lateral ventricle and the dentate gyrus (DG) of the hippocampus. They give rise to newborn neurons which integrate into pre-existing neuronal networks^11^. It has been shown that lipid metabolism affects NSPC activity: NSPC proliferation is regulated by fatty acid synthase (FASN)-dependent de novo lipogenesis^12^. Furthermore, the breakdown of lipids in mitochondria, known as fatty acid beta-oxidation (FAO), is an important regulator of embryonic^13^ and adult NSPC function^14,15^.

FAO and de novo lipogenesis are linked through lipid droplets (LDs), which are storage organelles for neutral lipids. LDs are mainly found in lipogenic tissues such as adipose and liver tissue; however, most cell types are able to form LDs when appropriately challenged^16^. In mammals, accumulation of LDs in cells of non-lipogenic tissues has so far been associated with diseased states such as cancer, obesity, or diabetes^17^. Rather than being simple storage compartments, recent data showed that LDs are highly dynamic organelles fulfilling crucial metabolic functions^18^ and there is emerging evidence that LDs might play an important role in the brain^19,20^. For instance, LDs in *Drosophila* and mouse astrocytes have been shown to be important in reaction to oxidative stress, protecting neurons from peroxidized lipids^21,22^. Similarly, the glial niche in *Drosophila* can support and protect neuroblasts by forming LDs under hypoxic conditions^23^. However, the role of LDs in mammalian NSPCs remains unknown. Using various approaches such as single-cell reconstruction, time-lapse analyses, single-cell RNA sequencing (scRNA-seq), metabolic measurements, manipulation of LD content and inhibition of several LD related pathways, we here show that LDs accumulate under physiological conditions and influence behaviour of adult mouse NSPCs.

## Results

### LDs are highly abundant in NSPCs under physiological conditions

**Fig. 1:**
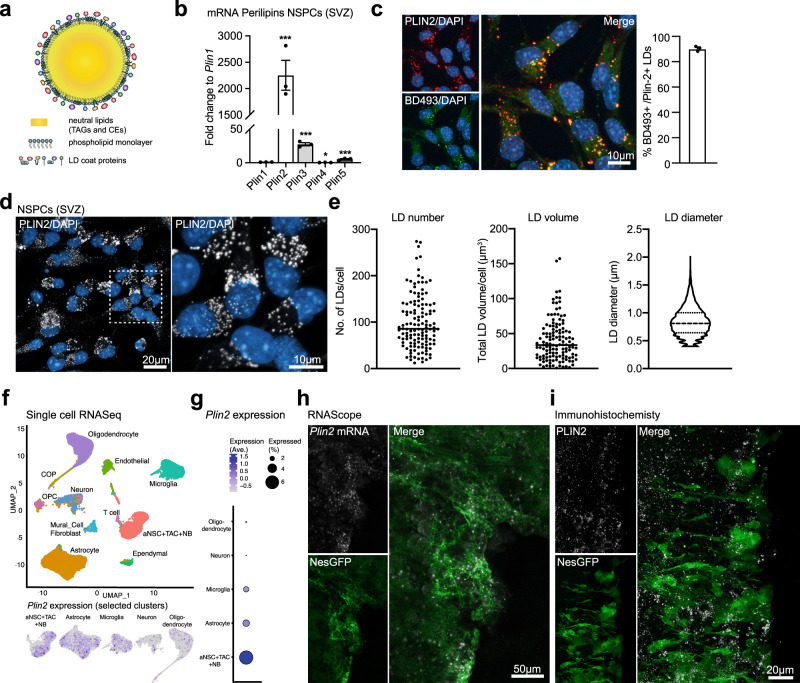
LDs are highly abundant in NSPCs under physiological conditions. **a** Schematic illustration of an LD, showing the TAG- and CE-containing core, surrounded by a monolayer of phospholipids and LD coat proteins. **b** qRT-PCR analysis showing that *Plin2* is the most abundant of the five *Plin* family members expressed in proliferating SVZ NSPCs (*n* = 3 samples, fold change versus *Plin1* ± SEM, one-way ANOVA with Dunnett’s multiple comparisons correction, adj. *p*-values: *Plin2* < 0.0001, *Plin3* = 0.0001, *Plin4* = 0.0113, *Plin5* = 0.0004). **c** Co-staining with the neutral lipid dye BD493 in proliferating SVZ NSPCs shows that >90% of BD493 positive LDs are also PLIN2-positive. Representative images are maximum intensity projections of the individual channels and a merge. (*n* = 520 cells from 3 coverslips, mean ± SEM). **d** Staining against PLIN2 (white) show a large variability in LD numbers and volume among proliferating SVZ NSPCs. Representative images are maximum intensity projections. Results were similar among 45 images from 3 coverslips. **e** Quantification of LD number, volume and diameter using 3D-single-cell reconstructions confirms the variability shown in **d**. (*n* = 130 cells from 3 coverslips). Each dot represents a single cell. LD diameters are shown as a violin plot. **f** Analysis of existing single-cell RNA-seq data^27,28^ from the adult SVZ niche cells shows that *Plin2* mRNA is expressed in astrocytes and NSPCs (the original nomenclature of the clusters was kept, thus here called aNSCs, TAC and NB). A UMAP showing unsupervised clustering and a feature plot for *Plin2* in astrocytes and NSPCs. **g** Expression analysis in the different clusters shows that *Plin2* expression is highest in NSPCs. **h** In situ hybridization using RNAscope shows *Plin2* expression (white) in GFP-positive cells (green) in the ventricular zone/SVZ of brain sections from NestinGFP mice. Representative maximum intensity projections. Results were similar among *n* = 3 mice. Nuclei (blue) are stained with DAPI. **i** Immunohistochemical staining against PLIN2 protein (white) in the SVZ of a NestinGFP mouse (green) shows that LDs are present in this region. Representative images are maximum intensity projections. Results were similar among *n* = 3 mice. Asterisks indicate the following *p*-values: * < 0.05; *** < 0.001.

LDs have a neutral lipid core containing triacylglycerols (TAGs) and cholesterol esters (CEs), which is surrounded by a phospholipid monolayer and decorated by LD coat proteins (Fig. 1a). One type of coat protein is the Perilipin (PLIN) family, containing five members (PLIN1-5). While PLIN2 and PLIN3 are ubiquitously expressed, the other members have a more tissue-specific expression^24^. We first assessed the expression of the *Plins* in proliferating adult SVZ and DG NSPCs in vitro by quantitative reverse transcription PCR (RT-qPCR). *Plin2* was the highest expressed *Plin* mRNA in NSPCs from both SVZ (Fig. 1b) and DG (Supplementary Fig. 1a). As PLIN2 is a highly specific LD coat protein^24^, antibodies against PLIN2 can be used to visualize LDs. To confirm that we visualized the majority of LDs by staining against PLIN2, we co-stained SVZ NSPCs with the fluorescent neutral lipid dye BODIPY493/503 (hereafter called BD493), which preferentially accumulates in LDs. Quantification of the co-localization with Plin2 showed that more than 90% of BD493 positive LDs were also positive for PLIN2 (Fig. 1c). Thus, these results validate PLIN2 as a robust marker of LDs in NSPCs.

We next characterized LD occurrence using high-resolution single-cell 3D-volume reconstructions (Supplementary Fig. 1b). Proliferating NSPCs from SVZ and DG showed a large variability in LD numbers, total LD volume and diameters of LDs, with neighbouring cells containing from a few LDs up to many hundreds of LDs (Fig. 1d, e; Supplementary Fig. 1c, d). Interestingly, DG NSPCs had lower numbers of LDs and smaller LD volume per cell (Supplementary Fig. 1d) than SVZ NSPCs, despite being grown under the same cell culture conditions as SVZ NSPCs, suggesting that LD accumulation in NSPCs is not only driven by nutrient availability. Theoretically, a cell can store the same number of lipids in a few large LDs or in many small LDs. Thus we tested whether NSPCs have a similar total stock of neutral lipids, despite the large differences in LD numbers. LD number per cell correlated with total LD volume in both NSPCs from SVZ and DG (Supplementary Fig. 1e), indicating that there is little compensation of lipid content in cells with low amounts of LDs. Cell size also could not explain the variability in LD accumulation, showing only a weak correlation with LD numbers (Supplementary Fig. 1f).

Proliferating NSPCs in vitro are not synchronized, thus cells fixed at a certain timepoint can be in any cell cycle stage. To address whether the observed variability in LD accumulation correlated with cell cycle stages, we used NSPCs isolated from the SVZ of adult FUCCI mice^25^. The FUCCI system allows for direct visualization of cell cycle stages due to two fluorescent proteins that fluctuate with cell cycle, leading to differently coloured nuclei (G1 red, S-phase entry yellow, S/G2/M green, non-coloured just after division). The same LD analysis using high-resolution single-cell 3D-volume reconstructions was performed in FUCCI NSPCs. LD numbers and volumes were also variable within each cell cycle stage analysed separately, thus could not per se explain the large LD variability among NSPCs (Supplementary Fig. 1g).

Taken together, these results show that there is a high variability in the availability of stored lipids in NSPCs grown under the same in vitro conditions, which cannot be explained by cell size or cell cycle stage. This suggests that among a putatively homogenous population of NSPCs, differences in lipid metabolic pathway activity exist, which might influence stem cell behaviour.

As NSPCs in vitro might show a different lipid metabolism than in their niche in the brain, we next addressed whether LDs are also present in NSPCs in vivo. Visualizing LDs in brain tissue is challenging, as the permeabilization required for antibody penetration affects LD stability^26^, and milder permeabilization approaches that can be used for immunohistochemistry in vitro have a poor antibody penetration in vivo. Thus, we investigated the presence of *Plin2* mRNA in vivo, using two different approaches: published scRNA-seq data and in situ hybridization. *Plin2* mRNA was clearly expressed in NSPCs and astrocytes in two recent scRNA-seq datasets^27,28^ of NSPCs and niche cells in the SVZ (Fig. 1f and g) and in the hippocampus (Supplementary Fig. 1h and i). To confirm the scRNA-seq data analysis, we used RNAscope technology, an advanced in situ hybridization assay, on brain tissue from NestinGFP reporter mice having GFP-labelled NSPCs^29^. *Plin2* mRNA was detectable in the SVZ (Fig. 1h and Supplementary Fig. 1j) and in the SGZ of the DG (Supplementary Fig. 1k and l), and co-localized with GFP-positive cells. Furthermore, PLIN2 immunostaining on brain sections from NestinGFP mice showed ring-like structures in NestinGFP-labeled cells in the ependymal layer/SVZ (Fig. 1i), and also within the DG (Supplementary Fig. 1m). Together, these data show that *Plin2* is also expressed under normal physiological conditions in NSPCs in vivo.

### LD accumulation changes significantly with quiescence

**Fig. 2:**
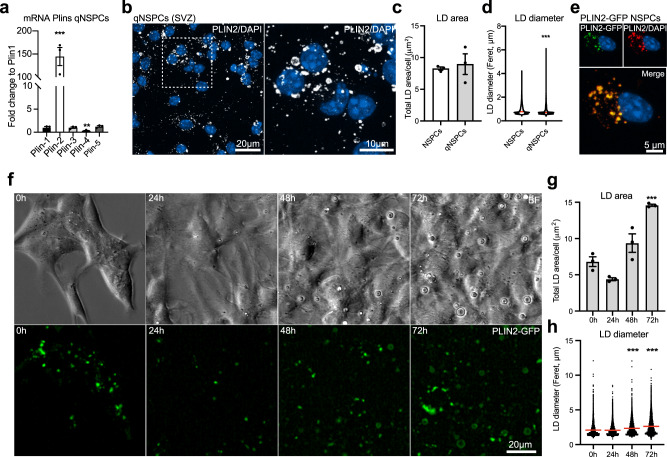
LD accumulation changes significantly with quiescence. **a** Analysis of mRNA expression by qRT-PCR shows that *Plin2* is the most abundant *Plin* family member in quiescent SVZ NSPCs (*n* = 3 samples, fold change versus *Plin1* ± SEM, one-way ANOVA with Dunnett’s multiple comparisons correction, adj. *p*-values: *Plin2* < 0.0001, *Plin4* = 0.0045). **b** Staining against PLIN2 (white) show that quiescent SVZ NSPCs also accumulate LDs, some of which have a larger size. Representative images are maximum intensity projections. Results were similar among 30 images from 3 coverslips per condition. DAPI-positive nuclei (blue). **c** The total LD accumulation did not significantly differ between proliferating and quiescent NSPCs (*n* = 378 proliferating cells, *n* = 567 quiescent cells from 3 coverslips per condition, mean ± SEM, Mann–Whitney test, two-tailed, *p* = 0.7). **d** Measurements of the diameter of LDs in proliferating and quiescent NSPCs revealed a significant increase in the proportion of large LDs with quiescence. Shown are violin plots depicting all LD diameters in the two conditions (Mann–Whitney test, two-tailed, *p* < 0.0001). **e** Stable overexpression of the *Plin2-Gfp* construct in NSPCs leads to GFP-positive LDs, as shown with PLIN2 co-staining. Representative images are maximum intensity projections. DAPI-positive nuclei (blue). Results were similar among 3 coverslips. **f** Time-lapse analysis of PLIN2-GFP NSPCs during quiescence induction. Representative brightfield (upper panel) and fluorescent images (lower panel) at 0, 24, 48 and 72 h after quiescence induction. Results were similar among 9 large fields of view from 3 wells. **g** Quantification of LDs over time revealed a significant increase in the number of LDs in quiescent NSPCs. (*n* = 3 wells, mean ± SEM, one-way ANOVA, Tukey’s multiple comparisons test, adj. *p*-value: 0 h vs. 72 h = 0.0003). **h** LD diameters were also significantly increased when full quiescence was reached. Violin plots depict all LD diameters at the timepoints analysed (one-way ANOVA, Tukey’s multiple comparisons test, adj. *p*-values: 0 h vs. 48 h < 0.0001; 0 h vs. 72 h < 0.0001). Asterisks indicate the following *p*-values: *** < 0.001.

NSPCs exist in either a quiescent or an activated state, which can be mimicked in vitro: addition of BMP4 in combination with a withdrawal of epidermal growth factor (EGF) leads to a quiescent state^30,31^. As several studies have shown that proliferating and quiescent NSPCs are metabolically different and that lipid metabolism is altered^15,32,33^, we next addressed whether LD accumulation differs under these two conditions. Similar to proliferating NSPCs, qRT-PCR revealed that *Plin2* was the highest expressed among the Plin family members in quiescent NSPCs (Fig. 2a), with comparable levels of *Plin2* mRNA between cell states (Supplementary Fig. 2a). In addition, *Plin1* and *Plin4* mRNA expression was significantly higher in quiescent NSPCs compared to proliferating NSPCs (Supplementary Fig. 2b). Co-staining of PLIN2 protein with BD493 showed that PLIN2 is a good LD marker in quiescent NSPCs (Supplementary Fig. 2c). However, the percentage of BD493 and PLIN2 double positive LDs was lower than in proliferating NSPCs (72.3% compared to 90.3%, Supplementary Fig. 2d and Fig. 1c), suggesting that other PLINs might play a role in LD regulation in quiescent NSPCs. While PLIN2-positive LD accumulation under quiescence did not significantly differ between proliferating and quiescent NSPCs (Fig. 2b and c), LDs appeared different, with some very large LDs in quiescent NSPCs (Fig. 2b). This observation was confirmed when measuring the diameter of LDs in proliferating and quiescent NSPCs, with a significant increase in the proportion of large LDs in quiescence (Fig. 2d). As LDs are sensitive to fixation and permeabilization, we next aimed to corroborate the observed differences in LDs using live cell imaging during quiescence induction of NSPCs. To do so, we generated a fluorescent LD-reporter system by stably overexpressing a *Plin2-Gfp* fusion construct in proliferating SVZ NSPCs, resulting in green fluorescent LDs. The LD-specific GFP signal was confirmed with co-staining against PLIN2 protein (Fig. 2e). As PLIN2 has been reported to stabilize LDs^34^, we first tested whether overexpression of PLIN2-GFP in proliferating NSPCs changed the observed variability of LDs using the same single-cell 3D reconstruction quantification as done in wild-type NSPCs. LD numbers and total LD volume were still variable among cells (Supplementary Fig. 2e and 2f), suggesting that the overexpression of this reporter construct did not fundamentally alter LD accumulation in NSPCs, although PLIN2-GFP NSPCs had slightly less LDs than non-transfected NSPCs (Supplementary Fig. 2f).

We next live-imaged PLIN2-GFP reporter NSPCs during the 72 h of quiescence induction (Fig. 2f and Supplementary Fig. 2g, supplementary movie 1). We used two different plating densities, with more than 2-fold differences in the initial number of cells, to control for potential confluency effects and contact inhibition effects. The area covered by PLIN2-GFP normalized to cell numbers showed a significant increase over the 72 h when full quiescence is reached, independent of cell density (Fig. 2g and Supplementary Fig. 2h). LD diameters were significantly increased at 48 h and 72 h after quiescence induction in the PLIN2-GFP NSPCs, (Fig. 2h). The large GFP-positive rings were confirmed to be LDs using a lipid dye (Supplementary Fig. 2i). Cell numbers doubled within the first 24h–48h of quiescence induction and then remained stable (Supplementary Fig. 2j), indicating that contact inhibition is not required for quiescence. Taken together, these data show that with quiescence, LDs increase in size. This suggests that quiescence is accompanied by a change in lipid metabolic pathways, in line with previously published gene expression and proteomic data^15,32,33^.

### Upon NSPC differentiation, neurons have significantly less LDs than astrocytes

**Fig. 3:**
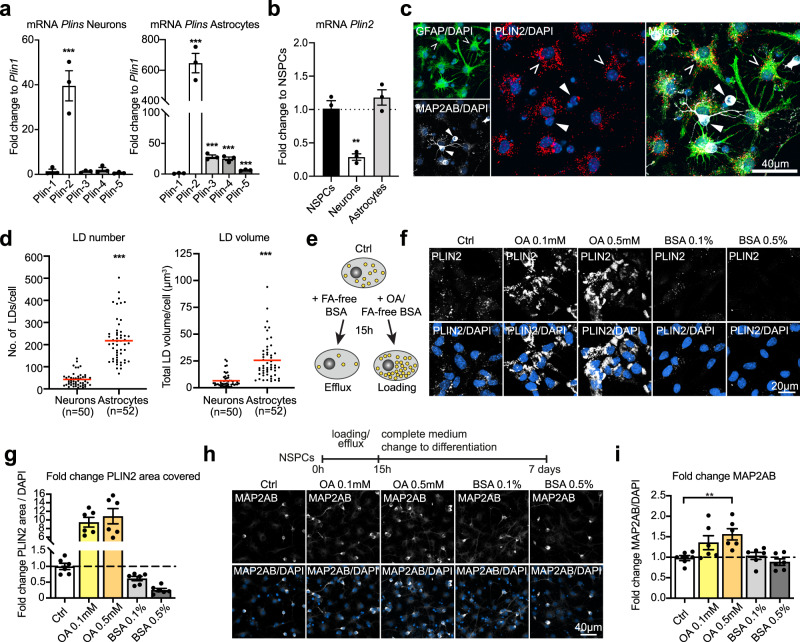
Neurons have significantly less LDs than astrocytes and artificially increasing LDs prior to NSPC differentiation leads to more neurons. **a** qRT-PCR shows that *Plin2* is the most abundant *Plin* member in neurons and astrocytes derived from SVZ NSPCs (*n* = 3 samples, mean fold change versus *Plin1* ± SEM, one-way ANOVA with Dunnett’s multiple comparisons correction, adj. *p*-values: neurons *Plin2* = 0.0002; astrocytes *Plin2* < 0.0001, *Plin3* < 0.0001, *Plin4* < 0.0001, *Plin5* = 0.0003). **b** NSPC-derived neurons express significantly less *Plin2* than NSPCs, while NSPC-derived astrocytes express amounts comparable to NSPCs (*n* = 3 samples, mean fold change ± SEM, one-way ANOVA with Dunnett’s multiple comparisons correction, prol vs neurons adj. *p*-value:0.0011). **c** NSPC-derived astrocytes (GFAP, green, broken arrowheads) contain many LDs (PLIN2, red) whereas NSPC-derived neurons (MAP2AB, white, filled arrowheads), contain less LDs. Representative images are maximum intensity projections. DAPI-positive nuclei (blue). Results were similar among 60 images from 3 coverslips. **d** 3D reconstruction of neurons and astrocytes revealed significantly increased LD numbers and total LD volume in astrocytes compared to neurons. (*n* = 50 neurons and *n* = 52 astrocytes from 3 coverslips, Mann–Whitney test, two-tailed, *p* < 0.0001). Each dot represents a cell, medians are shown in red. **e** Schematic illustration depicting the loading and efflux paradigm used. **f** OA-loading leads to a massive increase in LDs in proliferating SVZ NSPCs while fatty acid-free BSA reduces the LD accumulation. Representative images are maximum intensity projections. PLIN2 (white), DAPI-positive nuclei (blue). **g** Quantification of PLIN2 area covered with loading and efflux. Bar graphs represent mean ± SEM. (*n* = 6 coverslips from 2 experiments, Brown-Forsythe ANOVA, Dunnett’s multiple comparisons correction, adj. *p*-values: Ctrl vs. OA 1:100 = 0.0023; Ctrl vs. OA 1:20 = 0.0088; Ctrl vs. BSA 1:100 = 0.0398; Ctrl vs. BSA 1:20 = 0.0010). **h** Lipid loading at the NSPC stage influences the number of neurons (MAP2AB, white) formed after differentiation induction. Representative images are maximum intensity projections. DAPI-positive nuclei (blue). **i** Quantification of MAP2AB positive neurons shows a significant increase in the 0.5 mM OA condition compared to control NSPCs. Bar graphs represent mean ± SEM. (*n* = 6 coverslips from 2 experiments, one-way ANOVA, Holm-Sidak corrections, adj. *p*-value = 0.0057). Asterisks indicate the following *p*-values: ** < 0.01; *** < 0.001.

Besides being in an active or quiescent state, NSPCs can differentiate into astrocytes and neurons, and to a small extent also into oligodendrocytes. In vitro, differentiation occurs over five to seven days upon growth factor withdrawal and leads to a co-culture of astrocytes and neurons. To address expression of *Plin* family members in these NSPC-derived astrocytes and neurons separately, we established a separation protocol based on the different adherence properties of these two cell types (Supplementary Fig. 3a). We verified the enrichment of either neurons or astrocytes by analysing the expression of cell specific markers (Supplementary Fig. 3b) and analysed the expression of the *Plins* in these enriched fractions. For both astrocytes and neurons differentiated from NSPCs, *Plin2* remained the highest expressed *Plin* family member (Fig. 3a) and showed a strong co-localization (87.3% in astrocytes, 78.2% in neurons) with BD493 positive LDs (Supplementary Fig. 3c and d). However, direct comparison of *Plin2* mRNA showed that neurons had much lower *Plin2* mRNA levels than NSPCs and astrocytes (Fig. 3b). 3D reconstructions of individual neurons and astrocytes confirmed this finding: neurons had significantly less LD numbers and total LD volume and reduced variability (Fig. 3c and d) as compared to astrocytes, which were full of LDs and displayed a large variability in number and total volume of LDs. As neurons and astrocytes differ in cell size (Supplementary Fig. 3e), we next tested whether these differences persist if cell size was considered. Even when normalizing to cell volume, neurons had significantly less LD numbers (Supplementary Fig. 3f), meaning that they have significantly less stored neutral lipids available per cell than astrocytes.

Given the variability of LDs in NSPCs (Fig. 1d and e; Supplementary Fig. 1c and d) and the significant difference in LDs upon differentiation, we next addressed whether there was a correlation between the natural range of LD accumulation and fate choice. We used the PLIN2-GFP reporter NSPCs and categorized them into low, medium, and high GFP signal. Then, we followed them with time-lapse imaging over 5 days of differentiation to determine their fate. Each category of NSPCs gave rise to a similar proportion of astrocytes, neurons and cells that underwent cell death without significant changes between the categories (Supplementary Fig. 3g). These results suggest that initial numbers of LDs available are not sufficient to predict cell fate. However, the difference in LDs between astrocytes and neurons at the end of differentiation was still evident (Supplementary Fig. 3h and i), suggesting that lipid metabolism might be altered during or following fate choice.

### Artificially increasing LDs prior to NSPC differentiation leads to more neurons

We next tested whether we could influence fate by artificially increasing or decreasing LDs beyond the natural variability, prior to NSPC differentiation. Extracellular neutral lipids can be taken up by the cells and stored in LDs if not immediately used. Thus, we exposed proliferating NSPCs to two different doses of oleic acid coupled to fatty acid (FA) free BSA (0.1 mM and 0.5 mM) or FA-free BSA alone (0.1% and 0.5%) for 15 h (Fig. 3e). LD accumulation massively increased with oleic acid loading and led to a more than 9-fold increase in the area covered by PLIN2 compared to non-treated NSPCs (Fig. 3f and g). LDs decreased more than half with lipid efflux (Fig. 3f and g), demonstrating that LD content in NSPCs can indeed be artificially altered. Such treated NSPCs were then exposed to differentiation conditions. Analysis of the proportion of neurons after 7 days showed a significant increase of MAP2AB positive neurons in the loading condition (Fig. 3h and i). Remarkably, a significant increase in LD accumulation was observed in the loading condition even after 7 days of differentiation (Supplementary Fig. 3j and k), indicating that the initial lipid load in NSPCs was carried on with differentiation. To rule out that this effect was due to remaining oleic acid in the medium or attached to the plastic of the cell culture plate, proliferating NSPCs were treated for 15 h with oleic acid or FA-free BSA, followed by cell resuspension and two washing steps before the cells were plated into fresh cell culture dishes for differentiation. Under these conditions, the percentage of neurons was also significantly increased in oleic acid-loaded NSPCs (Supplementary Fig. 3l), pointing towards a cell intrinsic effect rather than remaining oleic acid in the medium. The increase in neurons was at least partially at the cost of astrocyte production, as the ratio of neurons (MAP2AB) to astrocytes (GFAP) was significantly changed in the oleic acid loading condition (Supplementary Fig. 3m). Taken together, these data suggest that artificially increasing LDs prior to NSPC differentiation leads to more neurons. Whether this is due to increased neuronal production, better survival of the newly generated neurons, a fate-influencing effect of oleic acid, or a yet unknown mechanism remains to be determined.

### The natural LD variability in NSPCs correlates with proliferative ability

**Fig. 4:**
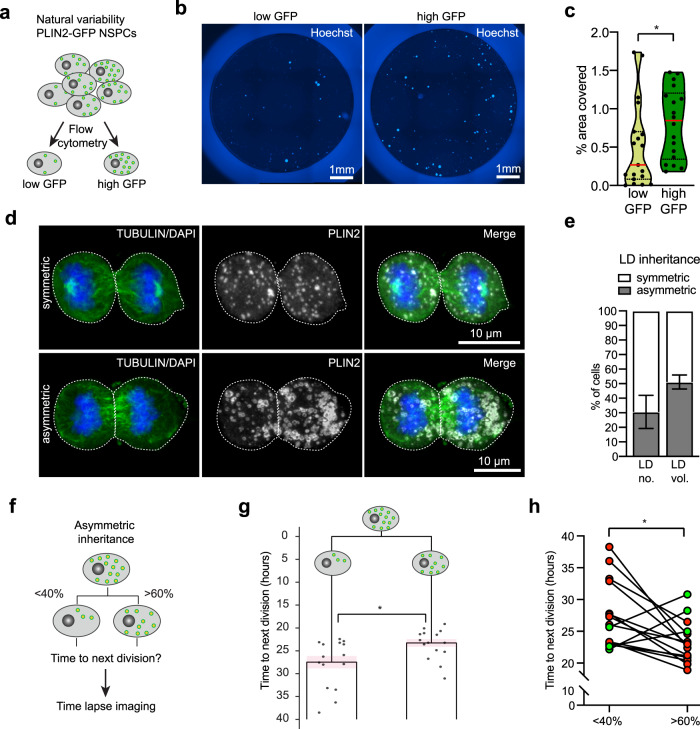
The total number of LDs as well as asymmetric LD inheritance influences NSPC proliferation. **a** Schematic illustration of the natural LD variability in PLIN2-GFP NSPCs and their sorting into a low and high GFP population. **b** High PLIN2-GFP NSPCs give rise to more progeny compared to low PLIN2-GFP NSPCs. Images show representative wells, cells are stained with live Hoechst (blue). Results were similar among 19 (low GFP) and 18 (high GFP) wells. **c** Quantification of the area covered by cells from the experiment shown in **b**. Dots represent individual wells. Medians are shown in red (*n* = 19 (low GFP) and 18 (high GFP) wells from 4 experiments, Mann–Whitney test, two-tailed, *p* = 0.031). **d** Paired-daughter cell analysis to study LD inheritance. Images represent maximum intensity projections of SVZ NSPC pairs, having either symmetric or asymmetric LD inheritance (PLIN2, white). The tubulin-positive mitotic spindle is shown in green, DNA (DAPI) is shown in blue. Results were similar among 3 coverslips. **e** Quantification of LD inheritance shows that one third of dividing NSPCs from the SVZ distributed the number of LDs asymmetrically (31.4%). The percentage of asymmetry even increased to half of all cells (51.4 %) when analysing LD volume. (*n* = 140 daughter cells (70 pairs) from 3 coverslips, mean ± SEM). **f** Schematic illustration of the time-lapse analysis to address the consequences of asymmetric LD inheritance. **g** Time-lapse imaging analysis of paired-daughter cells revealed a significant increase in time to next division in the daughter that asymmetrically inherited less LDs (23.3 h vs 27.5 h, *n* = 30 daughter cells (15 pairs), paired *t*-test, two-tailed, *p* = 0.0161). An average of all lineage trees is represented. Dots show the individual daughters at the time of next division, ±SEM is shown in light pink. **h** Individual paired-daughter cells from the time-lapse analysis and their time to next division are shown (*n* = 15 pairs, paired *t*-test, two-tailed, *p* = 0.0161). Red dots illustrate pairs for which the time to next division was shorter in the daughter that asymmetrically inherited more LDs. In three pairs, this was not the case (shown in green). Asterisks indicate the following *p*-value: * < 0.05.

While lipid loading or efflux allows to manipulate LD accumulation in NSPCs, it leads to massive LD changes beyond the natural variability, thus might not reveal more subtle influences of LDs on NSPC behaviour. We therefore next used the natural variability of LD content to probe if LDs correlated with the proliferative ability of NSPCs. We performed cell cycle analysis of proliferating PLIN2-GFP NSPCs using live Hoechst, and split the population retrospectively into the top 25% of PLIN2-GFP signal versus low 25% of PLIN2-GFP signal for analysis. This revealed that NSPCs with the top 25% of PLIN2-GFP signal had a significantly larger population of cells in S/G2/M phase than those with lower PLIN2-GFP signal (Supplementary Fig. 4a and b), suggesting that a higher number of LDs gives a proliferative advantage. Using fluorescent activated cell sorting (FACS) we next collected two populations of GFP-positive NSPCs, namely the ones containing the 25% highest and the 25% lowest PLIN2-GFP signal (Fig. 4a). Viability of these cells was confirmed using the viability marker Calcein red (Supplementary Fig. 4c). Following sorting, cells were cultured under proliferative conditions for 48 h. Interestingly, the collective PLIN2-GFP signal remained higher in the high GFP cells compared to the low-GFP cells, indicating that these two distinct populations remained stable over this time (Supplementary Fig. 4d and e). The high PLIN2-GFP NSPCs had an increased proportion of cells positive for the proliferation marker phospho-histone 3 (pH3), compared to the low PLIN2-GFP NSPCs (Supplementary Fig. 4d and f). Moderate proliferation effects, as seen at 48 h after sorting, might become more pronounced with prolonged time in culture. We thus sorted equal numbers of low and high GFP cells into 96-well plates at low density and let them grow as neurospheres for 7 days, after which the area covered by cells was assessed using the live nuclear dye Hoechst (Fig. 4b). This analysis revealed a significant increase in the area covered by cells in the high GFP NSPCs compared to the low-GFP NSPCs (Fig. 4c), suggesting that over a prolonged time in culture, proliferating NSPCs with high LD numbers generate more progeny. Taken together, this set of experiments suggest that within a population of NSPCs displaying a variable number of LDs, a higher number of LDs correlates with increased proliferative ability.

### LDs can be inherited asymmetrically during mitosis

Specified organelle distribution is critical during cell division and can drive downstream daughter cell behaviours^35^. However, little is known about the mitotic segregation and inheritance of LDs in general and specifically in NSPCs. To address this, we used high-resolution single-cell 3D-volume reconstructions of paired-daughter NSPCs (anaphase to telophase). As observed previously, LD variability was evident in paired-daughter cells, with pairs having from a few to many LDs, confirming that variability was not per se linked to cell cycle (Figs. 4d and S4h). Surprisingly, we observe many daughter pairs in proliferative conditions that displayed a clear asymmetric distribution of LDs among the two daughter cells (Figs. 4d and S4h). When defining asymmetry as at least a 1.5-fold difference between daughters, we found that 31.4% of dividing SVZ NSPCs distributed their LDs asymmetrically (Fig. 4d and e). The percentage of asymmetry increased to 51.4% when analysing total LD volume (Fig. 4e), suggesting that asymmetrical inheritance of LDs leads to unequal neutral lipid distribution among daughter cells. A more stringent analysis of asymmetric distribution for discrete values such as LD numbers, using the square root of n to determine the threshold of asymmetry, confirmed these results (Supplementary Fig. 4g). Similarly, dividing DG NSPCs showed an asymmetric distribution of LDs, with 40.1% of the cells having asymmetry in LD numbers and 56.1% asymmetry when considering total LD volume (Supplementary Fig. 4h–j). This asymmetric inheritance occurred in proliferative conditions, where both daughters will go on to remain as NSPCs, suggesting this asymmetric inheritance can occur in the absence of cell fate determinants.

### Asymmetric LD inheritance influences the time to the next division

To test whether such an asymmetric inheritance of neutral lipids is directly impacting NSPC behaviour, we next used the PLIN2-GFP reporter system to address whether asymmetric inheritance of LDs influences the time to next division. Asymmetrically dividing cells were followed individually until their next division using time-lapse microscopy (Fig. 4f). Intriguingly, asymmetric distribution of LDs led to a significant difference in the time to next division in the paired daughters, with cells that inherited more LDs dividing earlier than their sisters with less LDs (Fig. 4g and h, supplementary movie 2). Thus, these data show that asymmetric inheritance of LDs influences the proliferative behaviour of NSPCs and further confirms our finding that inheriting more LDs provides a proliferative advantage.

### There are few differences in gene expression between high and low LD-containing NSPCs

To better understand the differences between NSPCs with high and low amounts of LDs, we next performed scRNA-seq on sorted PLIN2-GFP NSPCs from the two categories used before, namely the 25% highest versus the 25% lowest PLIN2-GFP NSPCs. Cells were directly sorted into 384-well plates and a total of 304 cells passed all the subsequent quality controls. Low and high PLIN2-GFP NSPCs were dispersed among the 3 clusters determined by cell cycle phases, confirming that the number of LDs does not predict cell cycle phase (Supplementary Fig. 5a and b). As cell cycle was not a determining factor to cluster the two populations, it was regressed out for subsequent analyses (Supplementary Fig. 5c). Both low and high PLIN2-GFP NSPCs expressed comparable levels of NSPC markers such as fatty acid binding protein 7 (*Fabp7*), Vimentin (*Vim*), Nestin (*Nes*) and Hairy/Enhancer of Split 1 (*Hes1*), suggesting that both populations are indeed NSPCs (Supplementary Fig. 5d). *Plin2* gene expression levels were increased in high PLIN2-GFP NSPCs (Supplementary Fig. 5e), which also was confirmed by qRT-PCR on bulk sorted cells (Supplementary Fig. 5f). This indicates that increased PLIN2-GFP protein levels are also reflected by a change in *Plin2* mRNA on the gene expression level.

**Fig. 5:**
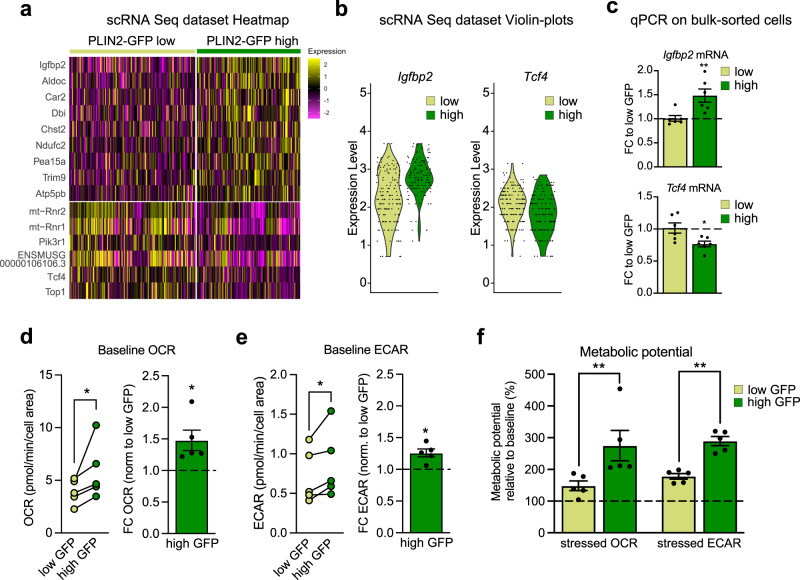
Single-cell gene expression analyses and functional metabolic measurements reveal differences between low and high LD-containing NSPCs. **a** scRNA-Seq analysis of low and high LD-containing NSPCs shows few differentially expressed genes. Heatmap of the significantly changed genes (Wilcoxon Rank Sum test, Bonferroni corrected for multiple testing, adj. *p*-value < 0.1). **b** *Igfbp2* and *Tcf4* are among the significantly altered genes. *Igfbp*2 is upregulated in high PLIN2-GFP NSPCs whereas *Tcf4* is downregulated. Violin plots of the scRNA-Seq dataset. **c** These changes are validated by qRT-PCR on bulk sorted low and high PLIN2-GFP cells (*n* = 6 sorted samples per condition from 2 independent experiments, mean fold change compared to low PLIN2-GFP ± SEM, unpaired *t*-test, two-tailed, *p*-value = 0.0053 *Igfbp2*; *p*-value = 0.0202 *Tcf4*). **d** Metabolic measurements of oxygen consumption rate (OCR) shows an increase in baseline OCR in high PLIN2-GFP NSPCs compared to low PLIN2-GFP NSPCs. The left graph shows individual OCR measures per experiment. The lines link low and high PLIN2-GFP samples from the same experiments. The right graph show the FC in OCR normalized to low PLIN2-GFP per experiment (*n* = 5 independent experiments shown as dots, bars indicate the mean value ± SEM, one-sample *t*-test on log2-transformed FC-values, two-tailed, *p*-value = 0.0208). **e** Metabolic measurements of the extracellular acidification rate (ECAR) shows an increase in baseline ECAR in high PLIN2-GFP NSPCs compared to low PLIN2-GFP NSPCs. The left graph show individual ECAR measures per experiment. The lines link low and high PLIN2-GFP samples from the same experiments. The right graph shows the FC in ECAR normalized to low PLIN2-GFP per experiment (*n* = 5 independent experiments, shown as dots, bars indicate the mean value ± SEM, one-sample *t*-test on log2-transformed FC-values, two-tailed, *p*-value = 0.0112). **f** Metabolic potential relative to baseline after inhibition of ATP synthase and removal of the proton gradient. The metabolic potential for OCR and ECAR is significantly increased in high PLIN2-GFP NSPCs compared to low PLIN2-GFP NSPCs (*n* = 5 independent experiments shown as dots, bars indicate the mean value ± SEM, two-way ANOVA, Holm-Sidak multiple comparison corrections, adj. *p*-value OCR = 0.0076; adj. *p*-value ECAR = 0.009). Asterisks indicate the following *p*-value: * < 0.05. ** < 0.01.

A small number of genes was significantly upregulated (9 genes) in the high vs low LD-containing NSPCs (Fig. 5a), such as insulin growth factor binding protein 2 (IGFBP2). Interestingly, IGFBP2 has been recently identified as a protein which is critically involved in promoting neural stem cell maintenance and proliferation^36^, suggesting that the high PLIN2-GFP NSPCs might have an advantage over the low PLIN2-GFP in keeping their stem cell potential. In line with this, the transcription factor 4 (TCF4, also called E2-2) was among the few significantly downregulated genes (6 genes) in the high PLIN2-GFP NSPCs (Fig. 5a). TCF4 has been shown to be important in neurogenesis, and neuronal maturation during development^37^. It also plays an important role in postnatal and adult neural stem cell differentiation^38^. While overexpression of TCF4 leads to increased neuronal differentiation, downregulation keeps the NSPCs in a more radial glial-like state^38^. Thus, the mild but significant transcriptional changes, with upregulation of a pro-stem cell gene (IGFBP2) and downregulation of a pro-differentiation gene (TCF4) in the PLIN2-GFP high NSPCs (Fig. 5a and b) suggest that the high PLIN2-GFP NSPCs are slightly more stem-like than the low PLIN2-GFP NSPCs. Both candidate genes were also confirmed to be altered by qRT-PCR on bulk-sorted low and high PLIN2-GFP NSPCs (Fig. 5c).

### Metabolic NSPC activity and capacity is increased with increased LD numbers

As LD accumulation likely reflects a certain metabolic state, we next performed functional metabolic measures using Seahorse technology to assess potential metabolic differences between low and high LD-containing NSPCs. Oxygen consumption rate (OCR), a measure for oxidative phosphorylation, and extracellular acidification rate (ECAR), a measure for glycolysis, were assessed in sorted low and high PLIN2-GFP NSPCs. Indeed, high PLIN2-GFP NSPCs had significantly higher baseline OCR (Fig. 5d) and ECAR (Fig. 5e) than low PLIN2-GFP NSPCs, indicating a general higher metabolic activity in high LD-containing NSPCs. Adding oligomycin, an ATPase inhibitor, allows the measurement of the capacity of cells to increase glycolysis. Adding FCCP, a proton gradient uncoupler, allows to assesses the maximal oxygen consumption. Both OCR and ECAR were significantly increased upon addition of oligomycin and FCCP in high PLIN2-GFP NSPCs compared to low PLIN2-GFP, showing that they have a higher metabolic capacity (Fig. 5f).

The differences in metabolic activity might come from differential FAO activity. We thus assessed the effect of the FAO inhibitor etomoxir on both low and high PLIN2-GFP NSPCs. Blocking FAO for 1 h resulted only in a mild decrease in OCR and a mild increase in ECAR in both NSPC populations (Supplementary Fig. 5g), indicating that FAO activity is not sufficient to explain the differences in the metabolic activity and capacity of low versus high PLIN2-GFP NSPCs.

To test whether manipulation of the LD content alters metabolic activity and capacity, we next sorted low and high PLIN2-GFP NSPCs and assessed their metabolic potential after overnight loading with a low dose of oleic acid. Indeed, such a lipid loading slightly increased the baseline OCR and the metabolic capacity of the low PLIN2-GFP NSPCs (Supplementary Fig. 5h and i). However, the effects of oleic acid loading were mild and did not reach statistical significance. Whether loading with a more complex lipid mixture would be able to increase metabolic activity and capacity in the low LD-containing NSPCs remains to be determined.

Taken together, while there is a clear metabolic difference between low and high LD-containing NSPCs, lipid availability and potential differences in FAO are not sufficient to explain these differences, suggesting that the underlying mechanisms are more complex.

### Higher ROS levels in the high LD-containing NSPCs do not lead to increased lipid peroxidation

**Fig. 6:**
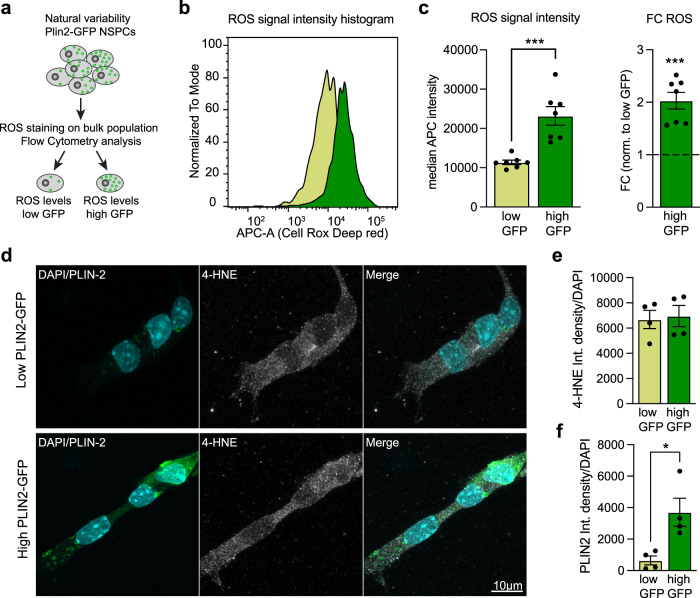
Higher ROS levels in the high LD-containing NSPCs do not lead to increased lipid peroxidation. **a** Schematic representation of the experiment to determine ROS levels. **b** ROS signal intensity histogram showing a clear difference between low and high PLIN2-GFP NSPCs. **c** Quantification of the median ROS intensity reveals that high PLIN2-GFP NSPCs have significantly higher ROS levels than low PLIN2-GFP NSPCs. Individual values (left panel) and fold change (right panel) normalized to the low PLIN2-GFP NSPCs are shown (*n* = 7 individual samples shown as dots, from 3 independent experiments, bars indicate the mean value ± SEM, raw values: Mann–Whitney test, two-tailed, *p*-value = 0.0006, FC-values: one-sample *t*-test on log2-transformed FC-values, two-tailed, *p*-value = 0.0001). **d** Staining against 4-HNE (white) shows no difference in this lipid peroxidation marker between proliferating low and high PLIN2-GFP NSPCs. Representative images are maximum intensity projections. **e** Quantification of the 4-HNE intensity per cell confirms that there are no differences (unpaired *t*-test, two-tailed, *p*-value = 0.8149) in the levels of 4-HNE between the two populations (*n* = 4 coverslips, from 2 independent experiments with a total of 126 high and 148 low PLIN2-GFP cells analyzed, bars indicate the mean value ± SEM). **f** Quantification of the PLIN2 intensity per cell confirms that the high PLIN2-GFP NSPCs have more LDs than the low PLIN2-GFP NSPCs (*n* = 4 coverslips, from 2 independent experiments with a total of 126 high and 148 low PLIN2-GFP cells analyzed, bars indicate the mean value ± SEM, unpaired *t*-test, two-tailed, *p*-value = 0.0167). Asterisks indicate the following *p*-value: * < 0.05. ** < 0.01. *** < 0.001.

Increased metabolic activity can be accompanied by increased generation of reactive oxygen species (ROS). To test whether the increased metabolic activity of high PLIN2-GFP NSPCs did generate more ROS, we used a fluorescent ROS sensor and analysed bulk populations of PLIN2-GFP NSPCs by flow cytometry (Fig. 6a, Supplementary Fig. 6a and b). When back-gating to the 25% highest and 25% lowest GFP NSPCs, a clear difference in ROS signal intensity was apparent, with the higher GFP NSPCs having significantly increased ROS levels compared to the lower GFP NSPCs (Fig. 6b and c). ROS can induce lipid peroxidation, which is toxic to cells. LDs in a *Drosophila* neural stem cell niche have recently been shown to have an antioxidant role, protecting lipids from lipid peroxidation^23^. To assess whether high LD-containing NSPCs were protected from lipid peroxidation, we used the lipid peroxidation product 4-hydroxynonenal (HNE) as readout. HNE is stably added to proteins and can be revealed with an antibody. Sorted low and high PLIN2-GFP NSPCs had comparable HNE staining intensity (Fig. 6d and e), despite the significant difference in ROS levels between the two populations (Fig. 6b and c). This suggests that the higher numbers of LDs in high PLIN2-GFP NSPCs (Fig. 6e and f) might at least partially protect them from ROS induced lipid peroxidation.

### Manipulating the breakdown or build-up of LDs decreases NSPC proliferation

**Fig. 7:**
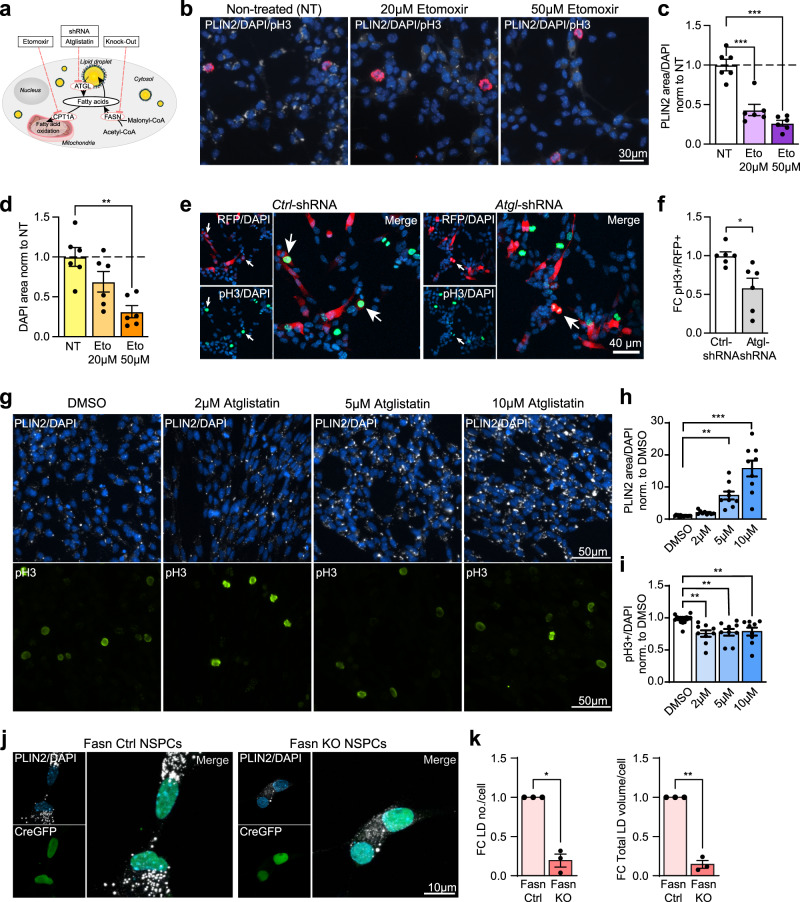
Manipulating the build-up or breakdown of LDs decreases NSPC proliferation. **a** Scheme illustrating the different manipulations. Carnitine palmitoyl transferase 1 (CPT1A), adipose triglyceride lipase (ATGL), fatty acid synthase (FASN). **b** Representative images of proliferating (pH3, red) NSPCs treated with Etomoxir. PLIN2 (white),DAPI (blue). Results were similar among 6 coverslips. **c** PLIN2 area covered, normalized to cell numbers, decreases with Etomoxir (*n* = 6 coverslips shown as dots, from 3 independent experiments, bars indicate the mean value ± SEM, one-way ANOVA, Holm-Sidak correction, *p*-value < 0.0001 for 20 and 50 μM Etomoxir). **d** The total DAPI area covered significantly decreased with Etomoxir (*n* = 6 coverslips shown as dots, from 3 independent experiments, bars indicate the mean value ± SEM, one-way ANOVA, Holm-Sidak correction, *p*-value = 0.066 for 20 μM and 0.0015 for 50 μM Etomoxir). **e** Representative images are maximum intensity projections of *Ctrl*-shRNA and *Atgl*-shRNA transfected proliferating NSPCs. DAPI (blue). Arrows indicate pH3+/RFP+cells. Results were similar among 6 coverslips per condition. **f** *Atgl* knockdown leads to a significant reduction in proliferating (pH3+) NSPCs compared to *Ctrl*-shRNA transfected NSPCs. (*n* = 6 coverslips from 2 independent experiments, bars indicate the mean ± SEM, unpaired *t*-test, two-tailed, *p*-value = 0.0113). **g** Representative images of proliferating NSPCs treated with Atglistatin. PLIN2 (white), pH3 (green), DAPI (blue). **h** PLIN2 area covered, normalized to cell numbers, significantly increases with Atglistatin (*n* = 9 coverslips shown as dots, from 3 independent experiments, bars indicate the mean ± SEM, one-way ANOVA, Holm-Sidak correction, *p*-value = 0.0018 for 5 μM and <0.0001 for 10 μM Atglistatin). **i** Proliferating pH3-positive NSPCs significantly decrease with Atglistatin (*n* = 9 coverslips shown as dots, from 3 independent experiments, bars indicate the mean ± SEM, one-way ANOVA, Holm-Sidak correction, *p*-value = 0.0022 for 2 μM, 0.0059 for 5 μM and 0.0094 for 10 μM Atglistatin). **j** PLIN2 (white) is reduced in *Fasn* KO NSPCs (positive for Cre-GFP, green) compared to Fasn Ctrl NSPCs. Representative images are maximum intensity projections. DAPI-positive nuclei (blue). **k** LD numbers and total LD volume significantly decrease in Fasn KO NSPCs (*n* = 3 coverslips, from 3 independent experiments with a total of 68 *Fasn* KO and 50 *Fasn* Ctrl cells analyzed, bars indicate the mean ± SEM, one-sample *t*-test on log2-transformed FC-values, two-tailed, *p*-value = 0.0972 and 0.026). Asterisks indicate the following *p*-value: ^+^ = 0.097. * < 0.05. ** < 0.01. *** < 0.001.

To further understand the role of LDs for NSPC behaviour, we next manipulated their breakdown or build-up, by inhibiting or knocking out several key players (Fig. 7a), and assessed the effects on LD numbers and proliferation.

We first assessed if the breakdown of LDs is required for FAO. To do so, we used two doses of the Cpt1a inhibitor etomoxir. Surprisingly, blocking FAO led to a significant reduction in LDs (Fig. 7b and c). This might be due to an increased lysis of LDs, as no fatty acids are reaching the mitochondria due to the Cpt1a blockage. While the ratio of NSPCs in S/G2/M phase assessed by pH3 did not significantly change (Supplementary Fig. 7a), we observed a significant reduction in the total number of NSPCs (Fig. 7c). This finding suggests that blocking FAO and concomitantly reducing LDs might affect survival and/or proliferation of NSPCs, as has been previously shown^14,15^. Further supporting a reduced survival or proliferation, the number of differentiated progeny after etomoxir treatment was strongly reduced (Supplementary Fig. 7b), while the number of neurons among the remaining cells was not affected (Supplementary Fig. 7c).

TAGs stored in LDs have to be broken down before they are available for use, for instance as an energy source or as membrane building blocks. Adipose triglyceride lipase (ATGL, also known as patatin-like phospholipase domain containing protein 2, PNPLA2*)* is a key lipase initiating the first step of TAG breakdown. To reduce the expression of this key lipase in proliferating NSPCs, we used a combination of shRNAs against *Atgl*. Indeed, in NSPCs expressing shRNAs against *Atgl*, LDs accumulated compared to NSPCs expressing a non-targeting control shRNA (Supplementary Fig. 7d and e). Proliferation was significantly lower after *Atgl* knockdown compared to control NSPCs (Fig. 7e and f). The same effect was seen when using the ATGL inhibitor Atglistatin: LDs accumulated in a dose-dependent manner (Fig. 7g and h), while proliferation was already significantly reduced with the lowest dose and was consistently lower in all doses tested (Fig. 7g and i). These data suggest that NSPCs need to access the TAGs in LDs through ATGL for proliferation. Interestingly, Atglistatin treatment during the first 2 days of differentiation initiation did not affect NSPC differentiation into neurons (Supplementary Fig. 7f, g and h), indicating that for differentiation initiation, access to LDs via ATGL is not critical. Whether they can access the LDs via lipophagy instead remains to be determined.

Lipids in LDs either originate from nutritional sources or from de novo lipogenesis. To assess the importance of de novo lipogenesis for LDs in NSPCs, we genetically ablated FASN in *Fasn* floxed NSPCs (*Fasn* KO) by virus mediated Cre-recombinase (Cre-GFP). *Fasn* KO NSPCs had significantly reduced LD numbers and total LD volume compared to control NSPCs (*Fasn* Ctrl) (Fig. 7j and k), suggesting that de novo lipogenesis is a major driver of LD formation in NSPCs. As previously reported^12^, *Fasn* KO NSPCs were significantly less proliferative than *Fasn* Ctrl NSPCs, as assessed by the proliferation marker pH3 (Supplementary Fig. 7i and j). These data are in line with the proliferation differences seen between low and high LD-containing NSPCs (Fig. 4b and c). Thus, reduced build-up of LDs by inhibiting de novo lipogenesis is associated with the reduced NSPC proliferation.

Taken together, these manipulations show that FAO and LDs are interconnected, that LDs can be broken down by ATGL in proliferating NSPCs, and that LDs in NSPCs store de novo produced lipids. Inhibiting these pathways leads to changes in LD accumulation in NSPCs, accompanied by a decrease in proliferation and/or survival, emphasizing the important role of LDs for NSPCs.

## Discussion

Our understanding of the importance of LDs for the cellular and systemic function of an organism has greatly increased over the last decade. It is now well accepted that LDs are versatile organelles which dynamically react to nutrient availability and whose turnover is based on a complex interplay between LD coat proteins and other intracellular proteins^16,18^. LDs are highly abundant in oocytes of vertebrates and invertebrates and are crucial for the proper development of the early embryo^39,40^, not only by providing lipids as an energy source, but also as carriers for excess proteins such as histones^41^. Surprisingly, despite their importance for early development, their role in stem cells remains poorly studied. Lipid metabolism has been shown to play an important role in the regulation of many somatic stem cells^42^. Thus, understanding the crucial role of these lipid metabolism organelles in stem cells is highly relevant to increase our knowledge of stem cell functioning.

We show here that LDs are highly abundant in adult mouse NSPCs under physiological conditions in vitro and provide evidence that LDs also occur in neurogenic niches in vivo (Fig. 1/Supplementary Fig. 1). As LDs are fragile organelles that can be disrupted by detergents commonly used for immunohistochemistry^26^, their presence in brain tissue has been underestimated thus far, except for sites such as the ependymal layer, which accumulate large amounts of LDs^43^. Our proof of LD accumulation in NSPCs in vivo is in line with data from recent single-cell datasets showing an important role for lipid metabolism in NSPCs^32,33^. However, given that NSPCs cultured in vitro encounter a different metabolic environment than NSPCs in their in vivo niche, the role of LDs in NSPCs in vivo remains to be further studied.

Interestingly, a specific lipid signal has been reported previously in rodent NSPCs in vitro and in vivo^44^, using proton nuclear magnetic resonance spectroscopy (1H-MRS). This lipid signal was also detected in the hippocampi of living humans^44^, where neurogenesis is ongoing throughout adulthood^45^. The specific 1H-MRS signal was shown to be caused by saturated and monounsaturated fatty acids, such as palmitic and oleic acid, which are usually stored in LDs. Although these findings were accompanied with technical concerns about the nature of the 1H-MRS signal detection^46^, the authors provided convincing evidence that this signal was correlating with NSPC properties^44^. It remains to be determined whether this lipid 1H-MRS signal could be related to LDs.

It previously has been shown that NSPC fate changes are accompanied by changes in metabolic profiles^12,47^. Thus, the differences in LD accumulation with different cell fates such as quiescence and differentiation (Figs. 2 and 3) might be a direct indicator of such metabolic changes. It will be interesting to address in more details the dynamics of LDs in correlation with cellular behaviour, and what happens to LDs upon manipulation of metabolic pathways. If there were a clear and closely timed correlation between metabolic pathway activity and LD abundance, observing dynamics of LDs might provide a way to predict cell behaviour.

Indeed, we here have explored a potential predictive power of LD numbers for fate choice or proliferation activity. While we did not see a direct correlation between the initial LD numbers in PLIN2-GFP NSPCs and the proportion of neurons and astrocytes formed, artificially increasing the numbers of LDs manyfold in NSPCs prior to differentiation increased the numbers of neurons formed (Fig. 3). This indicates that LDs in NSPCs are important for the differentiation process into neurons. However, the detailed mechanisms remain to be determined, as this artificially increased lipid availability could influence both fate and survival of the progeny, in line with previous publications showing that oleic acid triggers neuronal differentiation and axonogenesis^48,49^. We further show that within the natural variability of LD content, the number of LDs influences NSPC proliferation and that having more LDs provides an advantage for the cells (Fig. 4). Our scRNA-seq data further support these findings. The increased expression of IGFBP2 and the decreased expression of TCF4 in naturally high LD-containing NSPCs (Fig. 5) suggests that LD numbers might correlate with stemness in NSPCs. Both genes have been studied recently in the context of adult NSPCs and their relevance for maintaining stemness or promoting differentiation has been clearly demonstrated using knockdown and overexpression systems^36,38^. However, it is important to note that our observed differences in gene expression between high and low LD-containing NSPCs were small and that both populations express classical NSPC markers (Supplementary Fig. 5). These results suggest that even mild differences in the expression of these genes might ultimately determine stemness. Interestingly, it recently has been shown that increased LDs correlated with breast cancer progression and stemness of cancer stem cells^50^. As cancer stem cells and NSPCs share many lipid metabolic features^42^, LDs might also provide a readout for NSPC stemness. Furthermore, IGFBP2 has functional roles in growth and metabolism^51^. Whether the changes in IGFBP2 observed have a direct effect on adult NSPC metabolism remains to be determined.

Given the versatile function of LDs as lipid storage organelles, energy hubs, and detoxifying organelles as well as protein cargo organelles, further studies are required to reveal their detailed underlying mechanisms of action in NSPCs. We here show that within the natural variability of LD content, higher LD-containing NSPCs have increased metabolic activity and capacity (Fig. 5). While we did observe small changes in FAO, other metabolic changes might contribute to the overall increased metabolic activity. We further show that inhibiting LD breakdown negatively affects NSPCs (Fig. 7), suggesting that at least some of the effects are mediated through the LD content or LD coat protein availability.

How NSPCs sense the quantity of LDs they have available remains to be determined. Interestingly, Saito and colleagues showed that embryonic NSPCs lacking endogenous cholesterol biosynthesis were able to increase their supply of lipoproteins through the bloodstream and the ventricular fluid. This resulted in increased LD accumulation to compensate for the lack of cholesterol usually stored in LDs besides TAGs^52^, suggesting that NSPCs sense and regulate their lipid availability.

Asymmetric inheritance of proteins or organelles is an important mechanism for stem cells to differentially segregate cellular components that influence stem cell behaviour^53–56^. Our exciting findings of asymmetric LD inheritance and its functional consequence (Fig. 4) open up many new questions of how these organelles might influence stem cell proliferation. Further studies are required to address how this inheritance is regulated and what are the underlying mechanisms that provide a proliferative advantage to the daughter cell that inherits more LDs.

So far, accumulation of LDs in non-lipogenic tissue has been primarily associated with disease stages such as obesity and diabetes^57^. Interestingly, cancer cells, which are highly lipogenic, also accumulate LDs for as yet not fully understood reasons^58^. As we have previously shown, adult mouse NSPCs also have high levels of de novo lipogenesis^12^, similar to cancer cells. Thus, the observed accumulation of LDs in both cell types might be linked to this increased lipid synthesis to store the newly produced fatty acids and prevent lipotoxic consequences of such a high lipid synthesis. Indeed, the drastic reduction of LDs in *Fasn* KO NSPCs (Fig. 7) confirms that LDs in NSPCs are storing de novo produced lipids. As previously shown^12^, *Fasn* KO leads to decreased proliferation, which is in line with the lower proliferation of low LD-containing NSPCs.

Interestingly, several recent publications have demonstrated an important physiological role of LDs in glial cells. Bailey and colleagues showed that during *Drosophila* development, the niche glial cells can help protect neuroblasts from oxidative stress by forming LDs to shield polyunsaturated fatty acids coming from the neuroblasts from lipid peroxidation^23^. Similarly, Liu and colleagues showed that *Drosophila* neurons can shuttle lipids to glial cells upon elevated ROS and the glial cells then store those lipids in LDs. When disturbing this transfer, they observed neurodegeneration^21^. Along the same line, Ioannou and colleagues recently demonstrated that hyperactive mouse hippocampal neurons can transfer excess fatty acids to astrocytes, which in turn store them in LDs and consume them through FAO. This close metabolic interaction among neurons and astrocytes is thought to be neuroprotective and detoxifying. They further showed that this transfer of fatty acids is disturbed in mutations leading to Alzheimer’s disease^22^. Such a connection was also shown by Hamilton and colleagues: they observed aberrant accumulation of LDs in ependymal cells in an Alzheimer’s disease mouse model and showed that this aberrant accumulation in the neurogenic niche negatively impacted NSPC proliferation^59^. A protection from ROS induced lipid peroxidation might also play a certain role in adult mouse NSPCs: despite the increased metabolic activity and the increased ROS levels, lipid peroxidation was not different between high and low LD-containing NSPCs (Fig. 6), suggesting that LDs might provide a similar protection for unsaturated fatty acids as described in *Drosophila*^23^. Furthermore, a recent study has correlated ROS levels with NSPC stemness, showing that more stem-like NSPCs have higher ROS levels^60^. Our findings that high LD-containing NSPCs are metabolically more active, have higher ROS levels, and increased proliferation suggest that LDs might provide a general advantage for NSPCs and might be used as a readout to discriminated heterogenous NSPC populations.

In summary, our results demonstrate that LDs influence mouse NSPC behaviour. Together with other recent studies, these findings suggest that accumulation of LDs in glial and glia-like cells plays an important physiological role, which we are just starting to uncover.

## Methods

### Animals

C57/Bl6 male mice used for NSPC isolation were bought from Janvier (France). FUCCI Mice (B6.Cg-Tg(FUCCIS/G2/M)#504Bsi Tg(FUCCIG1)#596Bsi) were purchased through RIKEN (Japan). The Nestin-GPF mouse strain is the one originally described by Yamaguchi and colleagues^29^. Cells were extracted from 6–8-week-old male mice of the respective strains. All mice were kept under standard conditions in ventilated cages with ad libitum food and water. Housing conditions were as following: dark/light cycle 12/12, ambient temperature around 21–22 °C and humidity between 40 and 70% (55% in average). All experiments including animals were carried out in compliance with the Swiss law after approval from the local authorities (Cantonal veterinary office, Canton de Vaud, Switzerland).

### Plasmids

The GFP-Plin2 fusion construct was obtained from W. Kovacs (ETH Zurich). In brief, the GFP coding sequence was fused to the 5′ prime end (N-terminal side of the protein) of the mouse *Plin2* coding sequences, with a short linker sequence (tccggactcagatctcgagaa). This fusion construct was then cloned into a moloney leukemia virus backbone under the CAG promoter and virus was produced as previously described^61^.

Plasmids encoding for shRNAs against *Atgl* or control shRNA were bought from Origene (TF512624). Both plasmids contained an CMV-driven tRFP to visualize transfected cells.

### Cell culture

Adult mouse NSPCs from the SVZ and the hippocampus of 6–8-week-old C57/Bl6 male mice were isolated as following: Mice were shortly anaesthetized with isoflurane, followed by decapitation. Hippocampi and SVZ were micro-dissected and a single-cell suspension was generated using a GentleMacs Dissociator (Milteny) and the papain-based MACS Neural Tissue Dissociation Kit (Milteny, #130-092-628), according to the manufacturer’s instructions. Myelin removal was done using the MACs myelin removal beads (Milteny, #130-096-731) and a QuadroMACS Separator (Milteny, #130-090-976) according to the instructions. The resulting single-cell suspensions were cultured as neurospheres in DMEM/F12/GlutaMAX (Invitrogen, #31331-028) complemented with B27 (Invitrogen, #17504044), 20 ng/ml human EGF (AF-100-15, PeproTech), 20 ng/ml human basic FGF-2 (100-18B, PeproTech), and 1× PSF (Invitrogen, #15240062). Medium was changed every 2–3 days. The neurospheres were split a minimum of 5 passages to remove progenitors or other proliferating cells. The cells were then adapted to medium supplemented with N2 instead of B27, as described below (proliferation condition). Isolations of NSPCs were either done on individual mice or on pooled tissue. All the experiments were done on pooled NSPCs from 3–4 mice, up to passage 25. Cells were regularly controlled for expression of NSPC markers such as Sox2 and Nestin and for their ability to differentiate into neurons and astrocytes.

For NSPC propagation, cells were grown on uncoated standard plastic cell culture dishes (Corning, #430167, 100 × 20 mm, TC-treated). For experimental conditions, glass coverslips (10337423, Fisher), Lab-Tek chambers (LifeTechnologies, 155382PK) or standard plastic cell culture dishes were coated with poly-L-ornithine (Sigma, Cat. #P3655) and laminin (Sigma, #L2020-1MG) prior to cell plating.

#### Proliferation condition

Proliferating NSPCs were kept in DMEM/F12/GlutaMAX (Invitrogen, #31331-028) complemented with N2 (17502048, Gibco), 20 ng/ml human EGF (AF-100-15, PeproTech), 20 ng/ml human basic FGF-2 (100-18B, PeproTech), 5 mg/ml Heparin (H3149-50KU, Sigma) and 1× PSF (Invitrogen, #15240062). Medium was changed every 2–3 days.

#### Quiescence induction

Quiescence was induced as previously described^15^. In brief, 47.000 PLIN2-GFP NSPCs/cm^2^ (20.000 NSPCs/cm^2^ for sparse) were plated under proliferation conditions on coated plates and medium was changed after 24 h to quiescence medium containing DMEM/F12/GlutaMAX, N2, 20 ng/ml human basic FGF-2, 5 mg/ml Heparin, 50 ng/ml BMP4 (0.05 ng/ml, RnD Systems #5020-BP) and 1× PSF. Cells acquired a fully quiescent state 72 h after quiescence induction.

#### Differentiation condition

Proliferating NSPCs were plated on coated plates or glass coverslips in differentiation medium containing reduced amounts of growth factors. (The same medium as for proliferation condition, but only 2–4 ng/ml EGF and bFGF2). The medium was partially or fully changed after 2–3 days to medium without EGF and bFGF2. Cell were fixed or collected after 5–7 days of differentiation.

#### Atglistatin treatment

Atglistatin (Merck, SML1075) stock solution was prepared in dimethyl sulfoxide-DMSO (Sigma–Aldrich, D2438) at 70 mM and working stock solutions in medium at 1 mM. Cells were treated on coated glass coverslips for 48 h with final concentrations of 2, 5, or 10 μM for proliferation or at the beginning of differentiation (5 μM or 10 μM). Control cells were treated using DMSO only at a concentration corresponding to the highest concentration of Atglistatin.

#### Etomoxir treatment

Etomoxir (Sigma, #E1905) was dissolved in ddH2O to obtain a 10 mM stock solution. Proliferating NSPCs were treated for 48 h with 20 μM or 50 μM Etomoxir. For the differentiation experiments, etomoxir was added with the medium change to differentiation medium as described above and was left for 48 h. Medium was then replaced with medium containing no growth factors and no etomoxir was added for the remaining differentiation time.

#### PLIN2-GFP NSPCs

Wild-type SVZ NSPCs obtained as described above were infected with the moloney leukemia virus expressing the PLIN2-GFP construct described above. Infected NSPCs were expanded and enriched via FACSorting. A stable “line” was generated and used for several passages.

#### Fasn KO and Ctrl NSPCs

NSPCs from *Fasn* floxed mice^62^ previously isolated^12^, with the same method as described above, were a kind gift from S. Jessberger,. Ctrl wild-type NSPCs and Fasn KO NSPCs were infected with a CAG-Cre-GFP virus^12^ and fixed 4 days post-infection.

### Separation of differentiated cells

NSPC-derived co-cultures of neurons and astrocytes were harvested 7 days after differentiation induction (see “Cell culture”). Cell debris was gently washed off using the old medium and medium was discarded. Fresh medium was added to the plates, the neurons were rinsed off with a pipette boy, collected and centrifuged for 10 min at 300 × *g*. The remaining astrocytes were dissociated by incubation with TrypLE Express Enzyme (Gibco 12604013) at 37 °C for 6 min, collected with 1× PBS and centrifuged at 300 × *g* for 10 min. Pellets from both neurons and astrocytes were snap frozen on dry ice and used for RT-qPCR.

### Electroporation

Proliferating SVZ NSPCs were electroporated with plasmids encoding shRNA against Atgl or a scrambled control shRNA, using a 4D-Nucleofector X Unit EA (LZ-AAF-1002X, Lonza) and the P3 primary cell 4D-Nucleofector X solution (LZ-V4XP-3024, Lonza). Electroporated cells were plated in proliferation medium. Twenty four hours later, cells were trypsinized and plated on poly-L-ornithine/laminin coated coverslips. Forty eight hours after electroporation, cells were fixed, followed by immunohistochemistry.

### Time-lapse imaging experiments

All time-lapse experiments were done using a Nikon Ti2-E live cell microscope at 37 °C and 5.0% CO_2_ with a ×20 or ×40 objective. Cells were plated on poly-L-ornithine/laminin coated 4-well Lab-Tek chambers (LifeTechnologies, 155382PK) for imaging.

#### Asymmetric LD inheritance

29.400/cm^2^ PLIN2-GFP SVZ NSPCs were plated in proliferation medium. GFP and phase contrast images were acquired every 7 min for 72 h. Data processing and cells tracking were done manually using ImageJ (version 1.52, https://imagej.nih.gov/). Tree representation was done on a homemade phytree-based function of Matlab (R2019a, https://mathworks.com).

#### LD accumulation during quiescence induction

47.000 proliferating PLIN2-GFP SVZ NSPCs/cm^2^ (20.000 NSPCs/cm^2^ for sparse) were plated in proliferation medium. Medium was changed to quiescence medium (see “Cell culture”) after 24 h. After the medium change, GFP and phase contrast images were acquired every 20 min for 72 h. LD quantification at timepoint 0, 24, 48, and 72 h was done using Fiji. In brief, images were preprocessed by 8-bit conversion, “Enhance contrast”, “Gaussian blur” and “Subtract background”. Plin2 was the defined using the plugin MorphoLibJ and a mask was created. The mask was processed by “Fill holes” only to analyze total area of LDs and with “Fill holes and “Watershed” to analyze LD diameter. “Analyze particles” was used for the quantifications. Number of cells were manually counted on phase contrast images.

#### Differentiation condition

97,000 proliferating wild-type NSPCs/cm^2^ mixed with 1/10-1/20 PLIN2-GFP-positive NSPCs were plated in differentiation medium (see “Cell culture”) containing 1/5 growth factors. GFP and phase contrast images were acquired every hour during 5 days, starting 1 h after cells were plated. Cells were scored from 1 to 3 (1 being low LD content, 3 high LD content) in the starting frame by 4 independent researchers. A total score was calculated and cells were grouped into low LD content (scored 1–5), medium LD content (scored 6–10) and high LD content (scored 11-12). Cells were traced manually and fate determination was based on cell morphology combined with LD morphology.

### Cell cycle analysis

PLIN2-GFP NSPCs were incubated for 30 min with live Hoechst 33342 (Invitrogen, H1399), which integrates into the DNA in a linear manner and can be used to detect whether cells are in G1 (2× DNA) or S/G2 M (4× DNA) phase. NSPCs were analysed with a Cytoflex S Flow cytometer (Beckman Coulter). Data were analyzed using FlowJo software (FlowJo 10.6.2).

### FACS separation of PLIN2-GFP NSPCs

Proliferating SVZ PLIN2-GFP NSPCs were trypsinized and incubated for 10 min at 37 °C with the cell viability dye Calcein Red (C34851 ThermoFisher, 1:1000 in 1× PBS). Cells were washed twice with PBS and kept in EDTA-DPBS (E8008, Merck Millipore) at 4 °C during the sorting process. Cells were sorted on a MoFlo Astrios EQ cell sorter. Equal numbers of NSPCs with the 25% highest and lowest GFP signal were collected in proliferation medium, plated on glass coverslips and cultured for 48 h. For the 96-well plate experiments, 1000 NSPCs of the high or low category were directly sorted into the wells containing 150 µl proliferation medium and were cultured for 7 days and incubated with live Hoechst prior to fixation. For the 4-HNE analysis, low and high PLIN2-GFP NSPCs were sorted and plated on glass coverslips in proliferation medium. After attaching to the CS (max. 15 h), cells were fixed and used for immunohistochemistry.

### Lipid loading and efflux paradigm

#### Oleic acid and fatty acid-free bovine serum albumin stock solutions

A 12.5% fatty acid-free bovine serum albumin (A8806, Sigma, hereafter BSA) was prepared in water and subsequently sterile filtered. This solution was diluted to either 10% BSA using sterile filter 55 mM NaOH solution, aliquoted and stored at −20 °C until use, or further complexed with OA. For this, OA (O1008, Sigma) was dissolved in 55 mM NaOH at a final concentration of 50 mM and kept at 65 °C with frequent vortex to allow dissolution. Once dissolved, this OA solution was quickly sterile filter and then added to the 12.5% BSA solution to reach the final concentration of 10 mM OA in 10% BSA. Aliquots were then kept at −20 °C until further use.

Proliferating SVZ NSPCs were incubated with the appropriate concentration of BSA (0.1% or 0.5%) or OA (0.1 mM or 0.5 mM) for 15 h at 37 °C. Thereafter, they were fixed for immunohistochemistry or were subsequently plated under differentiation condition (see “Cell culture”) and fixed at day 7. For the differentiation experiments including the wash steps, NSPCs were collected after the 15 h of loading/efflux, spun down (300 × *g*, 3 min) and the pellet was washed 2× with PBS. These washed NSPCs were then plated under differentiation condition into fresh plates and fixed at day 7.

### Seahorse experiments

#### Baseline and stressed conditions

The Seahorse XF Cell Energy Phenotype Test Kit (Agilent, # 103325-100) was used, according to the manufacturer’s instructions, with a Seahorse XFe96 analyzer (Agilent) to measure the oxygen consumption rate (OCR) and extracellular acidification rate (ECAR) of NSPCs in a 96-well plate format. In brief, PLIN2-GFP NSPCs were sorted into the lowest (25%) and highest (25%) populations as described above. 50′000 cells were plated on coated Seahorse 96-well plates and left to attach overnight, maximum 15 h, in 80 μl normal proliferation medium per well. The next day, cells were washed 2× with XF DMEM assay medium (Agilent, #103575-100), supplemented with glucose (Agilent, 103577-100 Seahorse XF 1.0 M glucose solution), glutamine (Agilent, 103579-100 Seahorse XF 200 mM glutamine solution) and pyruvate (Agilent, 103578-100 Seahorse XF 100 mM pyruvate solution) according to the manufacturer’s instructions. Final concentrations: glucose 10 mM, glutamine 2 mM, pyruvat 1 mM. NSPCs where then incubated in the same XF assay medium including 20 ng/ml human EGF, 20 ng/ml human basic FGF-2 and 5 mg/ml Heparin for 1 h in a non-C02 but humidified 37 °C incubator (Agilent) before starting the assay. Baseline OCR and ECAR were measured over 3 timepoints (15 min). Oligomycine (1 µM) and FCCP (1 µM) were injected into each well using the Seahorse Cartridge system and OCR and ECAR measured immediately after.

#### Baseline and etomoxir effects

The same procedure was performed as described above. After baseline measurements, 20 μM etomoxir was injected into each well and OCR and ECAR were measured over 1 h.

#### Oleic acid loading effects

The same procedure as described above was performed on sorted low and high PLIN2-GFP cells that were either plated in normal proliferation medium or in medium supplemented with OA (1:500).

After completion of the assays, live Hoechst 33342 was injected in each well and incubated for 15 min to stain the nuclei. Cells were fixed with 2% PFA and images of the entire wells were taken with a Thunder microscope, ×5 objective. Area covered by the Hoechst signal was calculated using ImageJ and this value was used for normalization of the OCR and ECAR values of each well.

Each experiment was performed with 4–8 replicates per group and an average value of the replicates was calculated. Data were analyzed using Microsoft Excel. For the baseline, the 3rd measure (after 14.5 min) was used. For the metabolic capacity, the difference between the first measure after the oligomycin and FCCP injection and the baseline was calculated.

### ROS measurements

ROS measurements were performed with the CellROX Deep Red Flow Cytometry Assay Kit (ThermoFisher, # C10491) according to the manufacturer’s instructions. In brief, PLIN2-GFP NSPCs (200,000 cells per 24-well) were incubated with 500 nM Deep Red dye for 15 min at 37 °C. 1 µM SYTOX Blue Dead Cell Stain (ThermoFisher, # C10491) was added and incubated another 15 min at 37 °C. Cells were analyzed with a Cytoflex S Flow cytometer (Beckman Coulter), using 405-nm excitation for the SYTOX Blue Dead Cell stain and 635 nm excitation for the CellROX Deep Red reagent. Data were analyzed using FlowJo software (FlowJo 10.6.2). Single colour control samples were used for gating and NSPCs pretreated for 30 min with 0.1 mM TBHP served as a positive control.

### RT-qPCR

NSPC pellets from cultured or sorted cells were snap frozen on dry ice. RNA was extracted using RNeasy^®^ plus mini kit (Qiagen #74134) according to the manufacturer’s protocol, followed by cDNA preparation using the SuperScript™ IV First-Strand Synthesis System with oligo-dT primers (Invitrogen, #18091050). RT-qPCR was performed using TaqMan™ Fast Advanced Master Mix (#4444557 thermo fischer scientific) and Applied Biosystems TaqMan Assays, for the Perilipin family (see below). Power SYBR™ Green PCR Master Mix (#4367659, Fisher scientific) was used for analyzing the purity of the separated neurons and astrocytes, using primers for *Dcx, Tubb3 and Gfap* (as listed below). For validating candidate genes found by scRNA-seq, Power SYBR™ Green PCR Master Mix was used with KiCqStart SYBR green primers for *beta-actin*, *Igfbp2*, *Plin2*, and *Tcf4*.

Results were analyzed using the ddCT method, normalizing the samples to either eukaryotic *18* *S* rRNA or mouse *beta-actin*. Statistical analyses were performed on the dCT values.

### Primers

#### Plin1-5

- Mm00558672_m1
- Mm00475794_m1
- Mm04208646_g1
- Mm01272159_m1
- Mm00508852_m1

#### *Dcx, Tubb3, Gfap*, beta-Actin, Igfbp2, Plin2, Tcf4

- KiCqStart SYBR green Primers (KSPQ12012)
- *18S rRNA*
- 4333760 T
- *Beta-actin.*
- Mm01205647_g1

### Immunohistochemistry

Cells were fixed with 37 °C paraformaldehyde/PBS (4%) for 15–30 min at RT, washed 2× with PBS and were subsequently stored at 4 °C. All antibodies used and their dilution factor can be found in Supplementary Table 1.

#### Saponin-based staining protocol

The majority of LD stainings were performed as previously described, using the saponin-based protocol^26^. In short, cells were incubated in blocking buffer (1.5% Glycine, 3% BSA, 0.01% Saponin in 1× PBS) for 45 min, RT. Cells were incubated in primary antibody diluted in antibody diluent (0.1% BSA, 0.01% Saponin in 1× PBS) overnight, 4 °C. After 3 washes in 1× PBS, cells were incubated in secondary antibody diluted in antibody diluent for at least 1 h, RT, protected from light. Cells were washed once with 1× PBS. Thereafter incubated with DAPI (D9542, Sigma) diluted in 1× TBS for 10 min, followed by 2 washes in 1× TBS. Coverslips were mounted with a homemade PVA-DABCO-based mounting medium. When indicated, BODIPY™ 493/503 (Invitrogen, D3922, 1 mg/ml) staining was performed together with the secondary antibody incubation.

#### Triton-based staining protocol

Cells were incubated in blocking buffer (0.25% Triton X-100, 3% donkey serum in 1× TBS) for at least 30 min, RT, followed by incubation with primary antibodies in blocking buffer overnight, 4 °C. After 3 washes in 1× TBS, cells were incubated in secondary antibody in blocking buffer for at least 1 h, RT, protected from light. Cells were washed twice with 1× TBS. Thereafter incubated with DAPI diluted in 1× TBS for 10 min, followed by 1–2 washes in 1× TBS. Coverslips were mounted with a homemade PVA-DABCO-based mounting medium.

#### Wheat germ agglutinin (WGA) staining

After fixation, cells were incubated for 1 min with 10 µg/ml Wheat Germ Agglutinin Alexa Fluor™ 350 Conjugate in 1× HBSS. Cells were washed twice, using once 1× HBSS followed by 1× PBS for the second wash. Subsequently, cells were stained using the saponin-based protocol.

All the Antibodies used are listed in detail in Supplementary Table 1 provided in the supplementary information

### RNAScope

Twenty five micrometres sagittal brain sections from transcardially perfused NestinGFP mice were cut on a cryostat, mounted on SuperFrost+ slides (10149870, Fisher Scientific) and dried overnight. Hybridization was performed using the RNAscope 2.5 HD Detection Reagent—RED Kit (322360, Advanced Cell Diagnostics) according to the manufacturer’s instructions. Briefly, hybridization was performed for 2 h at 40 °C in a hybridization oven using branched DNA probes cocktail specific for murine *Plin2* mRNA (577111, Advanced Cell Diagnostics), followed by two washes in the provided wash buffer. Six amplification steps were performed subsequently at 40 °C with alternating incubations of 30 and 15 min and washes using the provided wash buffer. Sections were stained, using a triton-based protocol, against GFP as described under “Immunohistochemistry”.

### Single-cell RNA sequencing analysis of existing data

Single-cell RNA-Seq data were obtained from the original publications: Mizrak et al, accession number GEO: GSE109447, and Artegiani et al., accession number GEO: GSE106447, respectively. The data were then processed with Seurat 3.0 in R 3.6.1.The code used is available in the supplementary data.

### Single-cell RNA sequencing analysis of low and high PLIN2-GFP NSPCs

PLIN2-GFP NSPCs were prepared as described under FACS preparation to separate low and high PLIN2-GFP cells. SORT-seq: Single-cell mRNA sequencing was performed at Single Cell Discoveries (single-cell sequencing service, the Netherlands) according to an adapted version of the SORT-seq protocol^63^, with previously described primers^64^. Briefly, isolated cells were FAC-sorted into 384-well plates containing 384 primers and mineral oil (Sigma). After sorting, plates were snap frozen on dry ice and stored at −80 °C. For amplification cells were heat-lysed at 65 °C followed by cDNA synthesis using steps based on CEL-Seq2 protocol^65^. After second strand cDNA synthesis, barcoded material was pooled into libraries of 384 cells and amplified using IVT. Following amplification, library preparation was processed according to CEL-seq2 protocol where libraries were finally multiplexed using TruSeq small RNA primers (Illumina). Paired-end sequencing was achieved on Illumina Nextseq 500 (Read 1: 26 cycles, index read: 6 cycles, Read 2: 60 cycles).

scRNA-seq analysis: 26 base pairs from Read 1 were used for identification of the Illumina library barcode, cell barcode and UMI. 60 base pairs from Read 2 were assigned and aligned to the mouse genome (GRCm38) using the read-mapping algorithm Salmon^66^. Data were then demultiplexed to generate raw count tables. 304 cells passed quality controls based on mitochondria percentage <20% and doublets detection^67^. Raw counts were then normalized and scaled using Seurat (4.0.4) V3 single-cell analysis pipeline^68^. A cell cycle phase was further assigned to each single cell and then regressed out for downstream analysis. Single-cell clustering and differential gene expression were finally generated using standard parameters in Seurat pipeline.

### Image acquisition and analyses

All images used for LD characterizations, RNAScope and 4-HNE quantification were acquired with a confocal microscope (Zeiss, LSM 710, 780 and 900) with a ×40 or ×63 objective with digital zoom. 3-D single-cell reconstructions and the characterization of LDs were done with the imaging software Imaris (https://imaris.oxinst.com/) using the Volume and Spots modules of ImarisCell. Cell Volumes were created using the WGA channel for proliferating NSPCs, GFAP for astrocytes and MAP2AB for neurons. For NSPCs infected with *Ctrl* and *Atgl* shRNA virus, the RFP channel was used to create cell volumes. For the asymmetry analysis, daughter contours were drawn manually using the Volume module followed by LD detection using the automated Spots module. For the Fasn KO and Ctrl cells, only cells expressing Cre-GFP were considered for the quantification of their LDs per field of view by using the Imaris Spots module.

For the asymmetry analysis, daughter contours were drawn manually using the Volume module followed by LD detection using the automated Spots module.

Co-localization of BD493 and PLIN2 were assessed using Fiji (Version 2.0.0-rc-69/1.52p). In brief, BD493 acquisition was preprocessed using “Z-project”, “8-bit conversion”, “Subtract Background”, “Gaussian Blur” and “Brightness/Contrast”, “Set threshold” and converted to mask. BD493 was then quantified using “Analyze Particles”, and a mask was created. The BD493 mask was overlaid on the Plin2 acquisition, non-co-localized BD493 stained LDs were counted and subtracted from the total number of BD493 positive LDs. Number of cells was quantified through manual counting of DAPI-positive nuclei.

For quiescent NSPCs, LD quantification was not possible with Imaris due to the large ring-shaped LDs, which were not recognized by the Spot function. Thus, quantification of PLIN2 signal in quiescent NSPCs was done with Fiji, along with a set of images of corresponding proliferating. In brief, PLIN2 acquisition was preprocessed using “Z-Project”, “8-bit conversion”, “Gaussian blur” and “Subtract background”. The plugin MorphoLibJ was used to define the PLIN2 and a mask was created. The mask was processed with “Fill holes” and “Watershed”, number and diameter of PLIN2-positive LDs were quantified using “Analyze particles”. The number of cells was quantified through manual counting of DAPI-positive nuclei.

For proliferation and differentiation analyses, images were acquired with an epifluorescent microscope (Nikon 90i) using 20x or 40x objectives. Area covered by PLIN2 signal was measured following thresholding and counting of cells positive for the stained markers such as pH3 and MAP2AB was done in a blinded manner in a manual or semi-automatic way using ImageJ (version 1.52, https://imagej.nih.gov/). For the 96-well plate experiments, entire wells were imaged with a Thunder microscope (Leica) and stitched. Image analyses were done using ImageJ. Lipid peroxidation was assessed using 4-HNE signal on Fiji. Acquisition stacks were projected for maximum intensities; cell areas were drawn manually and stored in the ROI manager. Measurements of the 4-HNE and PLIN2 signal integrated densities were done without any prior thresholding, modification or adjustment of the signal.

For the etomoxir proliferation and differentiation experiments, the total number of cells was assessed using a tile image covering the entire coverslip. The images were quantified using the identical macro for all conditions. In brief, images were preprocessed using “Brightness/Contrast” before thresholding. Area covered of DAPI was then quantified using “Analyze particles” and normalized to the CS area quantified.

### Statistical analyses

Statistical analyses were performed with Prism (GraphPad) as following: for comparing two groups, a two-sided student *t*-test or a Mann–Whitney test was used,. When comparing 3 or more groups, a one-way ANOVA was used, followed by a Holm-Sidak’s multiple comparison test. Significance was considered for *p*-values < 0.05. For fold change (FC) analyses compared to a control group, FC-values were log2-transformed and a one-sample t-test was performed. The nature of the sampling (“n”), the statistical test and the exact *p*-values are described in each figure legends. A minimum of *n* = 3 is used for each statistical comparison.

### Reporting summary

Further information on research design is available in the Nature Research Reporting Summary linked to this article.

## Supplementary information

Supplementary Information

Description of Additional Supplementary Files

Supplementary Data 1

Supplementary Data 2

Supplementary Movie 1

Supplementary Movie 2

Reporting Summary

## Data Availability

The data supporting the findings from this study are available within the article file and its supplementary information. scRNA-seq raw and preprocessed data generated in this study have been deposited in the GEO database under accession code GSE187470. The following previously published datasets analyzed in this study were taken from the GEO database under accession code: GSE109447, and GSE106447. Any other raw data or non-commercial material used in this study are available from the corresponding author upon reasonable request. Source data are provided with this paper.

## Source data

Source Data

## Code availability

Details of the software codes used are described in the method section and are available from the corresponding author on reasonable request. Codes used to analyze scRNA-Seq data are provided as Supplementary Data 1 and Supplementary Data 2.
