# Supplementary material for: Lipid droplet availability affects neural stem/progenitor cell metabolism and proliferation
Source: Nat Commun. 2021 Dec 21;12:7362. doi: 10.1038/s41467-021-27365-7 (PMC8692608; doi:10.1038/s41467-021-27365-7)
Supplement: Supplementary file 1 — Supplementary Information [file 41467_2021_27365_MOESM1_ESM.pdf]

Supplementary Information

Lipid droplet availability affects neural stem/progenitor cell metabolism and proliferation

Mergim Ramosaj[1*], Sofia Madsen[1*], Vanille Maillard[1], Valentina Scandella[1], Daniel Sudria-Lopez[1], Naoya Yuizumi[2], Ludovic Telley[3] and Marlen Knobloch[1✉]

[1]Department of Biomedical Sciences, University of Lausanne, Lausanne, Switzerland. [2]Graduate School of Pharmaceutical Sciences, The University of Tokyo, Tokyo, Japan. [3]Department of Fundamental Neurosciences, University of Lausanne, Lausanne, Switzerland.
[*]These authors contributed equally.
[✉]marlen.knobloch@unil.ch

This file contains 7 supplementary figures, figure legends and a supplementary table

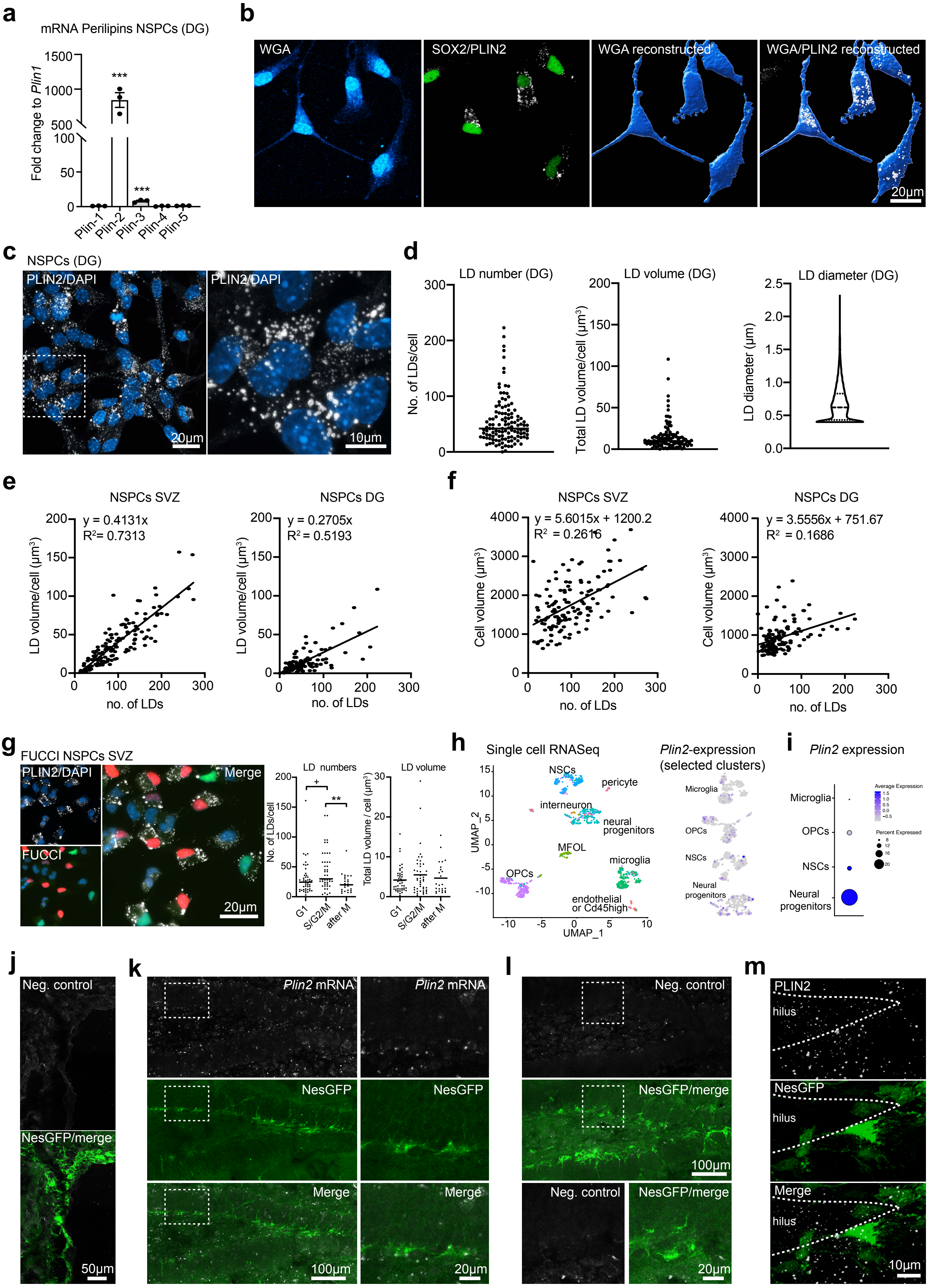

a
mRNA Perilipins NSPCs (DG)
Fold change to Plin1
***
***
Plin-1
Plin-2
Plin-3
Plin-4
Plin-5
b
WGA
SOX2/PLIN2
WGA reconstructed
WGA/PLIN2 reconstructed
20µm
c
NSPCs (DG)
PLIN2/DAPI
PLIN2/DAPI
20µm
10µm
d
LD number (DG)
No. of LDs/cell
LD volume (DG)
Total LD volume/cell (µm³)
LD diameter (DG)
LD diameter (µm)
e
NSPCs SVZ
y = 0.4131x
R²= 0.7313
LD volume/cell (µm³)
no. of LDs
NSPCs DG
y = 0.2705x
R² = 0.5193
f
NSPCs SVZ
y = 5.6015x + 1200.2
R² = 0.2616
Cell volume (µm³)
NSPCs DG
y = 3.5556x + 751.67
R² = 0.1686
g
FUCCI NSPCs SVZ
PLIN2/DAPI
Merge
FUCCI
20µm
LD numbers
No. of LDs/cell
LD volume
Total LD volume / cell (µm³)
G1
S/G2/M
after M
h
Single cell RNASeq
NSCs
pericyte
interneuron
neural progenitors
MFOL
microglia
OPCs
endothelial or Cd45high
UMAP_1
UMAP_2
Plin2-expression (selected clusters)
Microglia
OPCs
NSCs
Neural progenitors
i
Plin2 expression
Microglia
OPCs
NSCs
Neural progenitors
Average Expression
Percent Expressed
j
Neg. control
NesGFP/merge
50µm
k
Plin2 mRNA
Plin2 mRNA
NesGFP
NesGFP
Merge
Merge
100µm
20µm
l
Neg. control
NesGFP/merge
100µm
Neg. control
NesGFP/merge
20µm
m
PLIN2
hilus
NesGFP
hilus
Merge
hilus
10µm

Supplementary Figure 1: LDs are highly abundant in NSPCs, related to Figure 1. **a)** qRT-PCR shows that *Plin2* is the most abundant *Plin* expressed in proliferating DG NSPCs (n=3 samples, mean fold change versus *Plin1* +/- SEM, one-way ANOVA with Dunnett's multiple comparisons correction, p-values: *Plin2*<0.0001, *Plin3*=0.0002). **b)** 3D single cell reconstruction to analyse LD numbers, volumes and diameters. WGA (blue) outlines the cell, SOX2 marks the nucleus of NSPCs (green), PLIN2 visualizes LDs (white). Representative images are maximum intensity projections. **c)** PLIN2 (white) reveals a large variability in LD numbers and volume among neighbouring proliferating DG NSPCs. Representative images are maximum intensity projections. DAPI-positive nuclei (blue). Results were similar among 45 images from 3 coverslips **d)** Quantification of LD number, total LD volume and diameter confirms the variability shown in c. (n=120 cells from 3 coverslips). Each dot represents a cell. LD diameters are shown as a violin plot. **e)** LD numbers per cell correlate with total LD volume in NSPCs from SVZ and DG . **f)** Cell size weakly correlates with LD numbers in NSPCs. **g)** Proliferating FUCCI NSPCs, changing nuclear color with cell cycle, display a large variability of LDs (PLIN2, white) within each cell cycle stage. Representative images are maximum intensity projections. DAPI-positive nuclei (blue). Each dot represents a cell, medians are shown as a line. (One-way ANOVA, Tukey's multiple comparison test, G1 vs S/G2/M p-value=0.0506, after M vs S/G2/M p-value=0.008). **h)** Analysis of existing adult hippocampal niche single cell RNA seq data (Artegiani et al., 2017) shows that *Plin2* mRNA is expressed in NSPCs. UMAP of unsupervised clustering, and feature plot for *Plin2*. **i)** Expression analysis in the different clusters shows that *Plin2* is highest in NSPCs. **j)** *In situ* hybridisation using RNAscope shows no signal in the negative control in the ventricular zone/SVZ of Nestin-GFP mice. **k)** RNAscope shows *Plin2* expression in GFP positive cells in the DG in brain sections from Nestin-GFP mice. **l)** RNAscope negative control. **m)** PLIN2 (white) in the DG of a NestinGFP mouse (green) shows that LDs are present in this region. **For j-m:** Representative images are maximum intensity projections. Results were similar among n=3 NestinGFP mice. Asterisks indicate the following p-value: **<0.01. ***<0.001. + indicates a p-value of 0.0506.

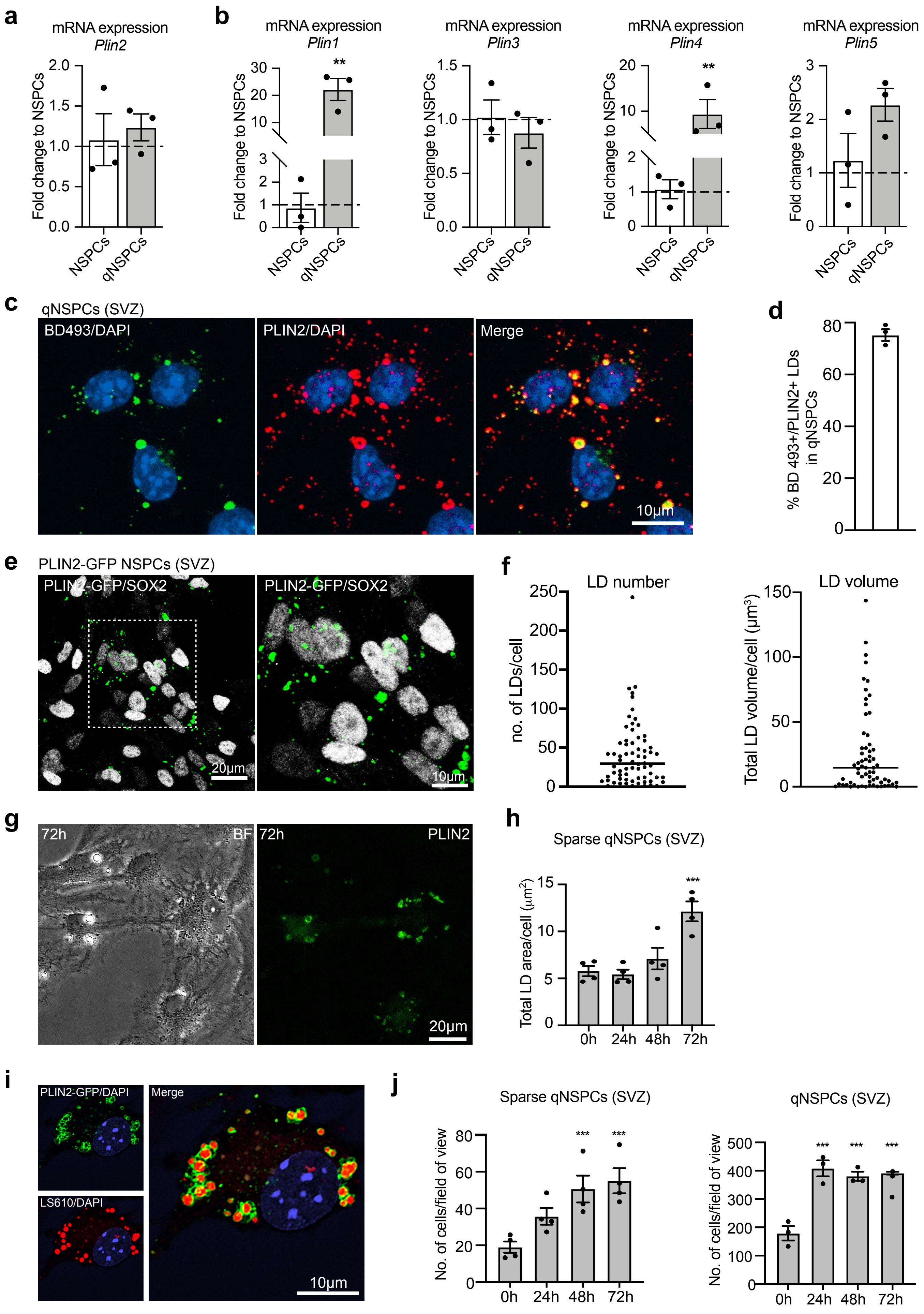
a
mRNA expression
Plin2
Fold change to NSPCs
NSPCs
qNSPCs
b
mRNA expression
Plin1
Plin3
Plin4
Plin5
**
c
qNSPCs (SVZ)
BD493/DAPI
PLIN2/DAPI
Merge
10µm
d
% BD 493+/PLIN2+ LDs in qNSPCs
e
PLIN2-GFP NSPCs (SVZ)
PLIN2-GFP/SOX2
20µm
10µm
f
LD number
no. of LDs/cell
LD volume
Total LD volume/cell (µm³)
g
72h
BF
PLIN2
20µm
h
Sparse qNSPCs (SVZ)
Total LD area/cell (µm²)
0h
24h
48h
72h
***
i
PLIN2-GFP/DAPI
LS610/DAPI
Merge
10µm
j
Sparse qNSPCs (SVZ)
qNSPCs (SVZ)
No. of cells/field of view

Supplementary Figure 2: LD accumulation changes significantly with quiescence, related to Figure 2.

a) qRT-PCR shows that *Plin2* is similarly expressed in proliferating and quiescent SVZ NSPCs (n=3 samples each condition, mean fold change +/- SEM, Mann-Whitney test, two-tailed, p=0.7). **b)** Among the other *Plin* family members, *Plin1* and *Plin4* were significantly upregulated with quiescence (n=3 samples each condition, mean fold change +/- SEM, unpaired t-tests, two-tailed, *Plin1* qNSPC vs NSPC p-value=0.0031, *Plin4* qNSPC vs NSPC p-value=0.0081). **c)** Co-staining of PLIN2 and BD493 shows co-localization of the two signals. Representative images are maximum intensity projections. DAPI-positive nuclei (blue). **d)** Quantification of the number of BD493/PLIN2 double positive LDs shows that 72.3% of BD493 positive LDs are also PLIN2 positive in quiescent SVZ NSPCs. (n=576 cells from 3 different coverslips, mean +/- SEM). **e)** Stable overexpression of a *Plin2-Gfp* fusion construct in proliferating SVZ NSPCs, leading to green fluorescent LDs. Representative images are maximum intensity projections. Nuclei are SOX2 positive (white). **f)** Quantification of the LD numbers and total LD volume in 3D reconstructed PLIN2-GFP NSPCs. Each dot represents a cell. **g)** Time-lapse analysis of sparsely plated PLIN2-GFP NSPCs during quiescence induction. Shown is a representative brightfield and fluorescent image at 72h after quiescence induction. **h)** Quantification of LDs over time revealed a significant increase in the number of LDs in sparsely plated quiescent NSPCs at 72h (n=3 wells, mean +/- SEM, one-way ANOVA, Tukey's multiple comparison test, p-value=0.0011 0h vs. 72h). **i)** PLIN2-GFP colocalises with the lipid dye Lipid Spot610 in quiescent PLIN2-GFP NSPCs, confirming that the large rings are LDs. Representative images are maximum intensity projections. **j)** Time-lapse analysis over 3 days of quiescence induction shows that NSPCs stop proliferating after 24h-48h. Bar graphs represent the mean +/- SEM of the number of cells per field of view at the indicated timepoints. (n=4 wells for sparse density, n=3 wells for normal density, one-way ANOVA, Tukey's multiple comparison test, p-value=0.0095 0h vs. 48h sparse, p-value=0.0036 0h vs. 48h sparse; p-value=0.0002 0h vs. 24h, p-value=0.0006 0h vs. 48h, p-value=0.0004 0h vs 72h). Asterisks indicate the following p-value: **<0.01. ***<0.001.

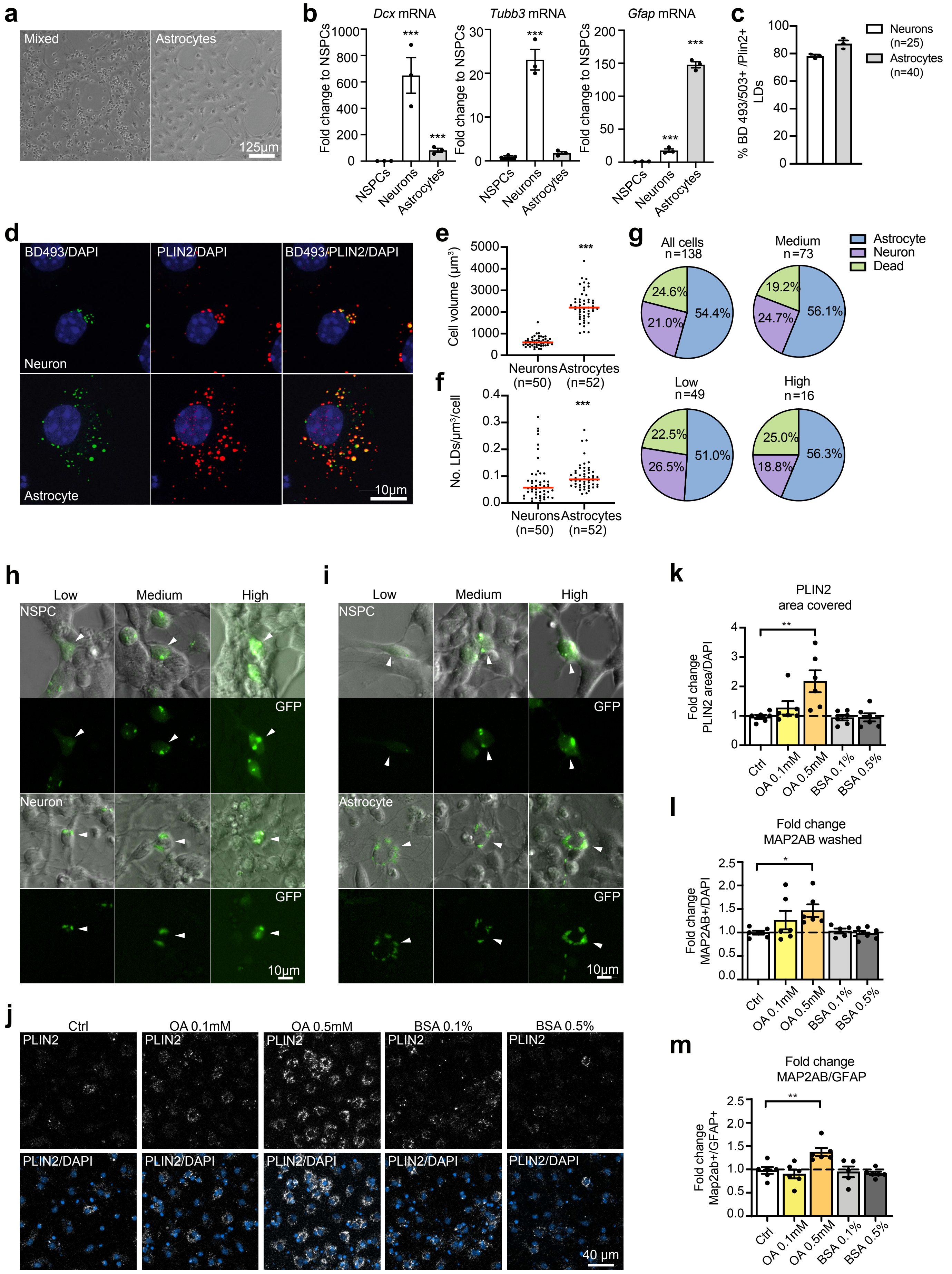

a
Mixed
Astrocytes
125µm
b
Dcx mRNA
Tubb3 mRNA
Gfap mRNA
Fold change to NSPCs
NSPCs
Neurons
Astrocytes
***
c
% BD 493/503+ /Plin2+ LDs
Neurons (n=25)
Astrocytes (n=40)
d
BD493/DAPI
PLIN2/DAPI
BD493/PLIN2/DAPI
Neuron
Astrocyte
10µm
e
Cell volume (µm³)
Neurons (n=50)
Astrocytes (n=52)
f
No. LDs/µm³/cell
g
All cells n=138
Medium n=73
Low n=49
High n=16
Astrocyte
Neuron
Dead
24.6%
21.0%
54.4%
19.2%
24.7%
56.1%
22.5%
26.5%
51.0%
25.0%
18.8%
56.3%
h
Low
Medium
High
NSPC
GFP
Neuron
10µm
i
Astrocyte
k
PLIN2 area covered
Fold change PLIN2 area/DAPI
Ctrl
OA 0.1mM
OA 0.5mM
BSA 0.1%
BSA 0.5%
l
Fold change MAP2AB washed
Fold change MAP2AB+/DAPI
m
Fold change MAP2AB/GFAP
Fold change Map2ab+/GFAP+
j
PLIN2
PLIN2/DAPI
40 µm

Supplementary Figure 3: Neurons have significantly less LDs than astrocytes and artificially increasing LDs prior to NSPC differentiation leads to more neurons, related to Figure 3.

a) Representative image before and after separation of differentiated NSPC progeny. Results were similar among n=3 samples per condition. **b)** qRT-PCR shows enrichment of neuronal genes (doublecortin (*Dcx*), beta 3 tubulin (*Tubb3*)) in the neuronal fraction, whereas the astrocytic marker glial fibrillary acidic protein (*Gfap*) is enriched in the astrocytic fraction (n=3 samples per condition, mean fold change +/- SEM, one-way ANOVA with Dunnett's multiple comparisons correction, *DCX* NSPCs vs. neurons and NSPCs vs. astrocytes p-value<0.0001; *Tubb3* NSPCs vs. neurons p-value<0.0001; *Gfap* NSPCs vs. neurons and NSPCs vs. astrocytes p-value<0.0001). **c)** Out of BD495/503 positive LDs, the majority are PLIN2 positive in astrocytes (87.3%) and neurons (78.2%). (n=25 neurons and n=40 astrocytes from 3 coverslips). Dots represent individual cells; the median is shown in red. **d)** Representative images are maximum intensity projections of a neuron and astrocyte co-stained for PLIN2 (red) and BD493/503 (green). Nuclei are blue. **e)** Cell volumes are significantly different between neurons and astrocytes. Dots represent individual cells; the median is shown in red. (n=50 neurons and 52 astrocytes from 3 coverslips, Mann-Whitney test, two-tailed, p-value<0.0001). **f)** LD numbers are still significantly higher in astrocytes when normalized to cell volume. Dots represent individual cells; the median is shown in red. (n=50 neurons and 52 astrocytes from 3 coverslips, Mann-Whitney test, two-tailed, p-value<0.0004). **g)** No significant difference in the proportion of cells becoming astrocytes, neurons or dying was observed among the low, medium and high PLIN2-GFP NSPCs in a differentiation time-lapse experiment. (n=138 individually followed cells from 4 wells from 2 experiments). **h and i)** Representative images of low, medium and high PLIN2-GFP NSPCs at beginning of differentiation (upper panel, arrowhead) and after becoming a neuron or an astrocyte respectively (lower panel, arrowhead). Results were similar among n=4 wells. **j)** Lipid loading of NSPCs leads to increased LDs (PLIN2, white) after differentiation. Nuclei are blue. **k)** Area covered by PLIN2 significantly increases in the 0.5mM OA condition. Bar graphs represent mean fold change +/- SEM. (n=6 coverslips from 2 experiments, one-way ANOVA, Holm-Sidak correction, p-value=0.0017). **l)** MAP2AB positive neurons also increased when lipid loaded or -effluxed NSPCs were washed prior to differentiation. Bar graphs represent mean fold change +/- SEM. (n=6 coverslips from 2 experiments, one-way ANOVA, Holm-Sidak correction, p-value=0.0317). **m)** The ratio of MAP2AB positive neurons to GFAP positive astrocytes increased with OA loading (n=6 coverslips from 2 experiments, bar graphs represent mean +/-SEM, one-way ANOVA, Holm-Sidak correction, p-value=0.0087).

Asterisks indicate the following p-value: *<0.05. **<0.01. ***<0.001.

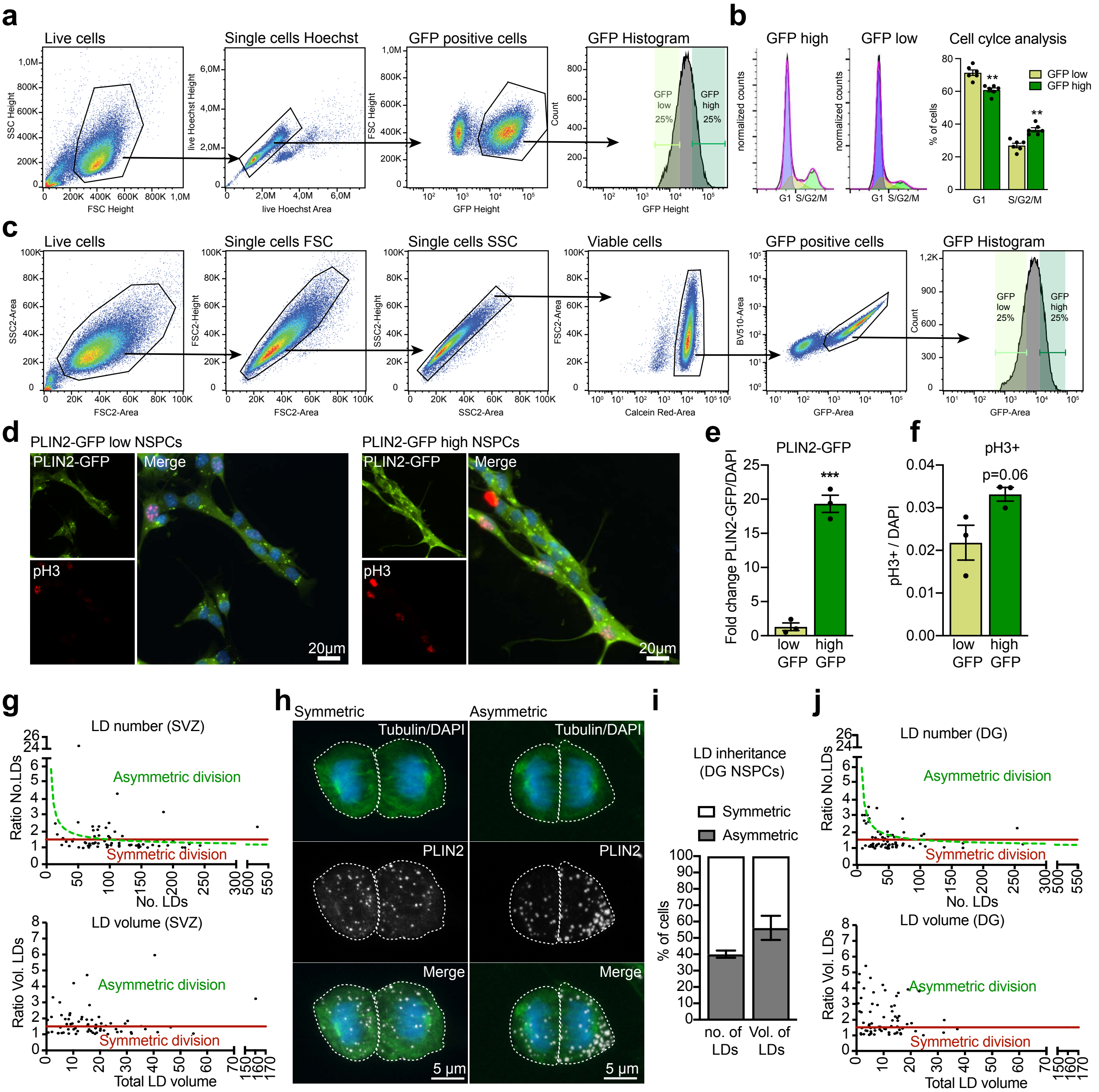
a
Live cells
Single cells Hoechst
GFP positive cells
GFP Histogram
SSC Height
FSC Height
live Hoechst Height
live Hoechst Area
FSC Height
GFP Height
Count
GFP low 25%
GFP high 25%
b
GFP high
GFP low
Cell cylce analysis
normalized counts
G1 S/G2/M
% of cells
GFP low
GFP high
G1
S/G2/M
c
Live cells
Single cells FSC
Single cells SSC
Viable cells
GFP positive cells
GFP Histogram
SSC2-Area
FSC2-Area
FSC2-Height
SSC2-Height
SSC2-Area
FSC2-Area
Calcein Red-Area
BV510-Area
GFP-Area
Count
GFP low 25%
GFP high 25%
d
PLIN2-GFP low NSPCs
PLIN2-GFP high NSPCs
PLIN2-GFP
Merge
pH3
20μm
e
PLIN2-GFP
Fold change PLIN2-GFP/DAPI
***
low GFP
high GFP
f
pH3+
pH3+ / DAPI
p=0.06
low GFP
high GFP
g
LD number (SVZ)
Ratio No.LDs
Asymmetric division
Symmetric division
No. LDs
LD volume (SVZ)
Ratio Vol. LDs
Asymmetric division
Symmetric division
Total LD volume
h
Symmetric
Asymmetric
Tubulin/DAPI
PLIN2
Merge
5 μm
i
LD inheritance (DG NSPCs)
Symmetric
Asymmetric
% of cells
no. of LDs
Vol. of LDs
j
LD number (DG)
Ratio No.LDs
Asymmetric division
Symmetric division
No. LDs
LD volume (DG)
Ratio Vol. LDs
Asymmetric division
Symmetric division
Total LD volume

Supplementary Figure 4: The total number of LDs as well as asymmetric LD inheritance influences NSPC proliferation, related to Figure 4.
a) Cell cycle analysis of proliferating PLIN2-GFP SVZ NSPCs. Representative flow cytometry plots illustrate the gating strategy. **b)** Representative cell cycle profiles of GFP high and GFP low cells and the quantification of cells in G1 and S/G2/M. (n=6 samples from 3 experiments, paired t-test, two-tailed, p-value=0.0015). **c)** Flow cytometry-based separation of GFP low and GFP high PLIN2-GFP SVZ NSPCs. Representative flow cytometry plots illustrate the gating strategy. **d)** Proliferating GFP low and GFP high PLIN2-GFP NSPCs keep their GFP expression pattern. Epifluorescent images of the individual channels and a merge are shown. Mitotic cells are shown in red (pH3), Nuclei (blue) are stained with DAPI. **e)** Quantification of the area covered by PLIN2-GFP 48h after sorting showed a significant difference between the low GFP and high GFP cells (mean fold change +/- SEM, n=3 coverslips per condition, unpaired t-test, two-tailed, p-value=0.0002). **f)** Quantification of the number of pH3+ cells revealed a trend of increased proliferation in the GFP high NSPCs. (mean +/- SEM, n=3 coverslips per condition, unpaired t-test, two-tailed, p-value=0.0606). **g)** Paired-daughter cell analysis to study LD inheritance in SVZ NSPCs. Each cell is displayed as a dot. The red line represents the 40/60 ratio used (=1.5), for the LD numbers, the green dotted line represents the more stringent square root of n analysis. **h)** Representative images are maximum intensity projections of DG NSPC pairs in anaphase, having either symmetric (left panel) or asymmetric (right panel) LD inheritance (white). The mitotic spindle (green) is revealed with tubulin staining, DNA is stained with DAPI (blue). Results were similar among 3 coverslips. **i)** Quantification of LD inheritances shows that 40.1% of dividing NSPCs from the DG distributed the number of LDs asymmetrically. The percentage of asymmetry increased to 56.1 % when analysing total LD volume. (n=146 daughter cells (73 pairs) from 3 coverslips, mean +/- SEM). **j)** The same display of the paired-daughter cell analysis as shown in **h** shown here for the DG NSPCs.
Asterisks indicate the following p-value: **<0.01. ***<0.001.

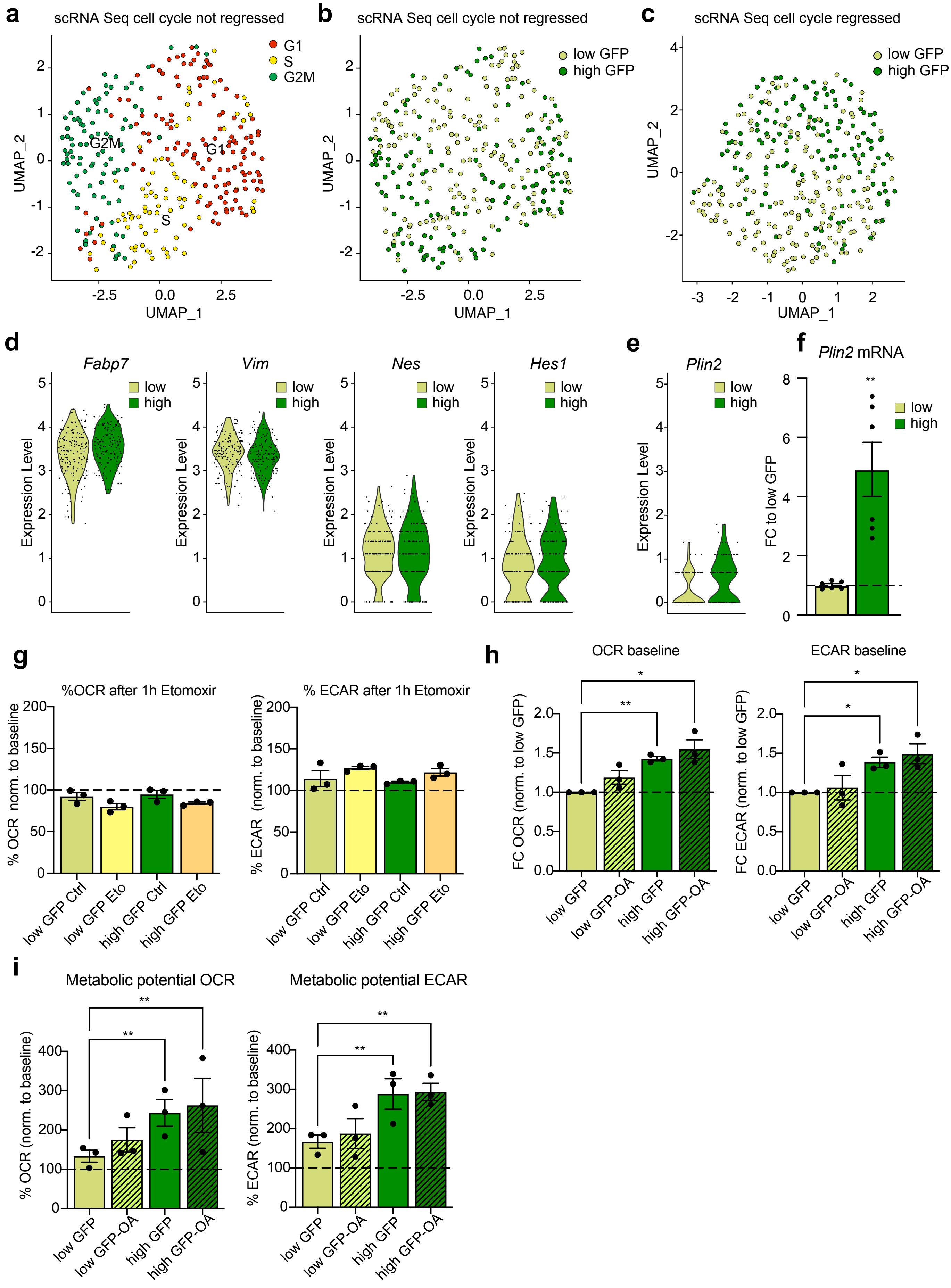
a
scRNA Seq cell cycle not regressed
G1
S
G2M
UMAP_1
UMAP_2
b
scRNA Seq cell cycle not regressed
low GFP
high GFP
c
scRNA Seq cell cycle regressed
d
Fabp7
Vim
Nes
Hes1
low
high
Expression Level
e
Plin2
f
Plin2 mRNA
FC to low GFP
g
%OCR after 1h Etomoxir
% OCR norm. to baseline
% ECAR after 1h Etomoxir
% ECAR (norm. to baseline)
low GFP Ctrl
low GFP Eto
high GFP Ctrl
high GFP Eto
h
OCR baseline
FC OCR (norm. to low GFP)
ECAR baseline
FC ECAR (norm. to low GFP)
low GFP
low GFP-OA
high GFP
high GFP-OA
i
Metabolic potential OCR
% OCR (norm. to baseline)
Metabolic potential ECAR
% ECAR (norm. to baseline)

Supplementary Figure 5: Single cell gene expression analyses and functional metabolic measurements reveal differences between low and high LD-containing NSPCs, related to Figure 5.

a) scRNA Seq analysis comparing low and high LD-containing NSPCs shows that cells group together by cell cycle stage. Shown is a UMAP of all cells, G1 (red), S (yellow) or G2/M (green). **b)** The same UMAP as in a, displaying low and high PLIN2-GFP NSPCs, confirms that the number of LDs *per se* does not predict cell cycle phase. **c)** UMAP after cell cycle regression shows a regrouping of the low and high PLIN2-GFP NSPCs into two groups. **d)** Violin plots of the known NSPC markers fatty acid binding protein 7 (*Fabp7*), Nestin (*Nes*), Hairy/Enhancer of Split 1 (*Hes1*) and Vimentin (*Vim*) show similar expression in both populations. **e)** Violin plot of *Plin2* shows higher *Plin2* in the high PLIN2-GFP NSPCs. **f)** Confirmation of increased *Plin2* mRNA expression in bulk-sorted high PLIN2-GFP NSPCs, supporting that increased PLIN2-GFP protein levels are accompanied by increased *Plin2* mRNA levels (n=6 sorted samples per condition from 2 independent experiments, mean fold change +/- SEM, unpaired t-test, two-tailed, p-value<0.0001). **g)** Changes in OCR and ECAR after 1h treatment with the FAO inhibitor etomoxir. The OCR decrease and ECAR increase in low and high PLIN2-GFP NSPCs did not reach statistical significance (n=3 independent experiments, individual experiments are shown as dots, bars indicate the mean value +/-SEM, two-way ANOVA, non-significant). **h)** Pre-treatment of NSPCs with OA has only a mild and non-significant effect on OCR at baseline (left) and no effect on ECAR (right). The general differences in baseline OCR and ECAR between low and high PLIN2-GFP NSPCs are also confirmed in this experiment (n=3 independent experiments, individual experiments are shown as dots, bars indicate the mean value +/-SEM, two-way ANOVA, non-significant). **i)** Similarly, the metabolic potential in OCR is only mildly and non-significantly increased after OA (left) and has no effect on ECAR (right), whereas the difference between low and high PLIN2-GFP NSPCs is clearly confirmed (n=3 independent experiments, individual experiments are shown as dots, bars indicate the mean value +/-SEM, two-way ANOVA, Holm-Sidak correction, OCR p-value=0.0387 low GFP vs. high GFP; p-value=0.0294 low GFP vs. high GFP OA; ECAR p-value=0.007 low GFP vs. high GFP; p-value=0.007 low GFP vs. high GFP OA). Asterisks indicate the following p-value: *<0.05. **<0.01.

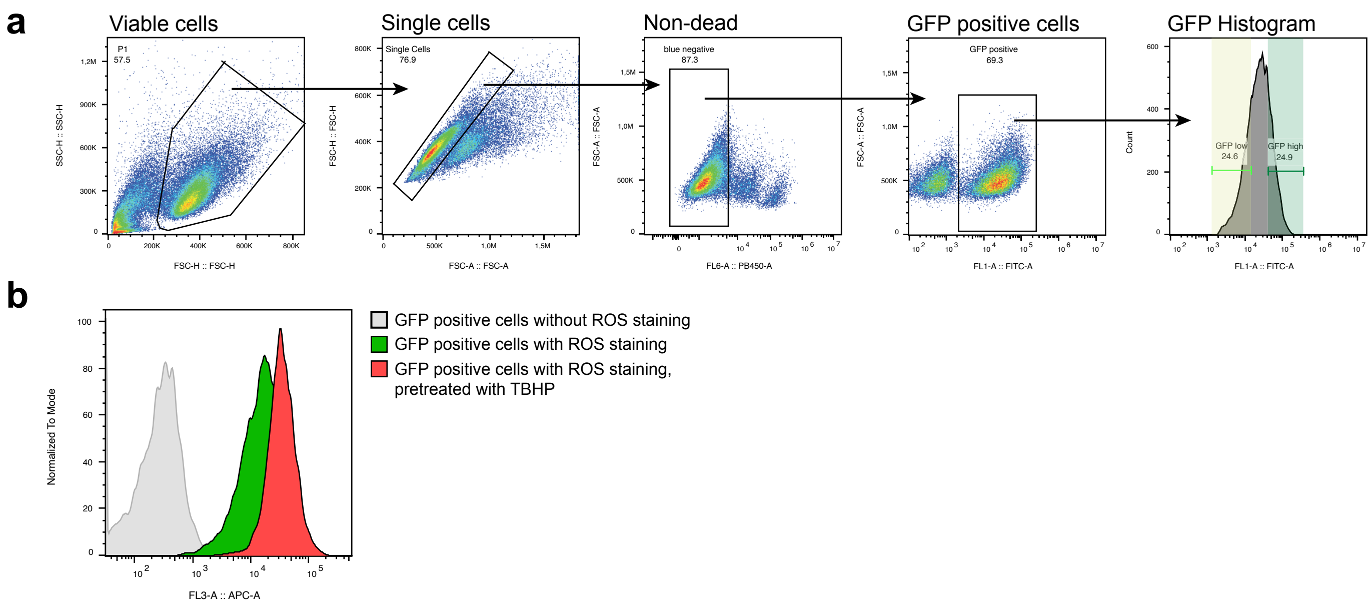
a
Viable cells
Single cells
Non-dead
GFP positive cells
GFP Histogram
P1
57.5
Single Cells
76.9
blue negative
87.3
GFP positive
69.3
GFP low
24.6
GFP high
24.9
SSC-H :: SSC-H
FSC-H :: FSC-H
FSC-H :: FSC-H
FSC-A :: FSC-A
FSC-A :: FSC-A
FL6-A :: PB450-A
FSC-A :: FSC-A
FL1-A :: FITC-A
Count
FL1-A :: FITC-A
b
GFP positive cells without ROS staining
GFP positive cells with ROS staining
GFP positive cells with ROS staining, pretreated with TBHP
Normalized To Mode
FL3-A :: APC-A

Supplementary Figure 6: Higher ROS levels in the high LD-containing NSPCs do not lead to increased lipid peroxidation, related to Figure 6.

a) ROS level analysis of proliferating PLIN2-GFP SVZ NSPCs. Representative flow cytometry plots illustrate the gating strategy. **b)** ROS intensity histogram of an unstained PLIN2-GFP sample (grey), a stained sample (green) and a sample pretreated with TBHP (red), which increases ROS levels. These controls confirm that the CellROX Deep red dye works as expected.

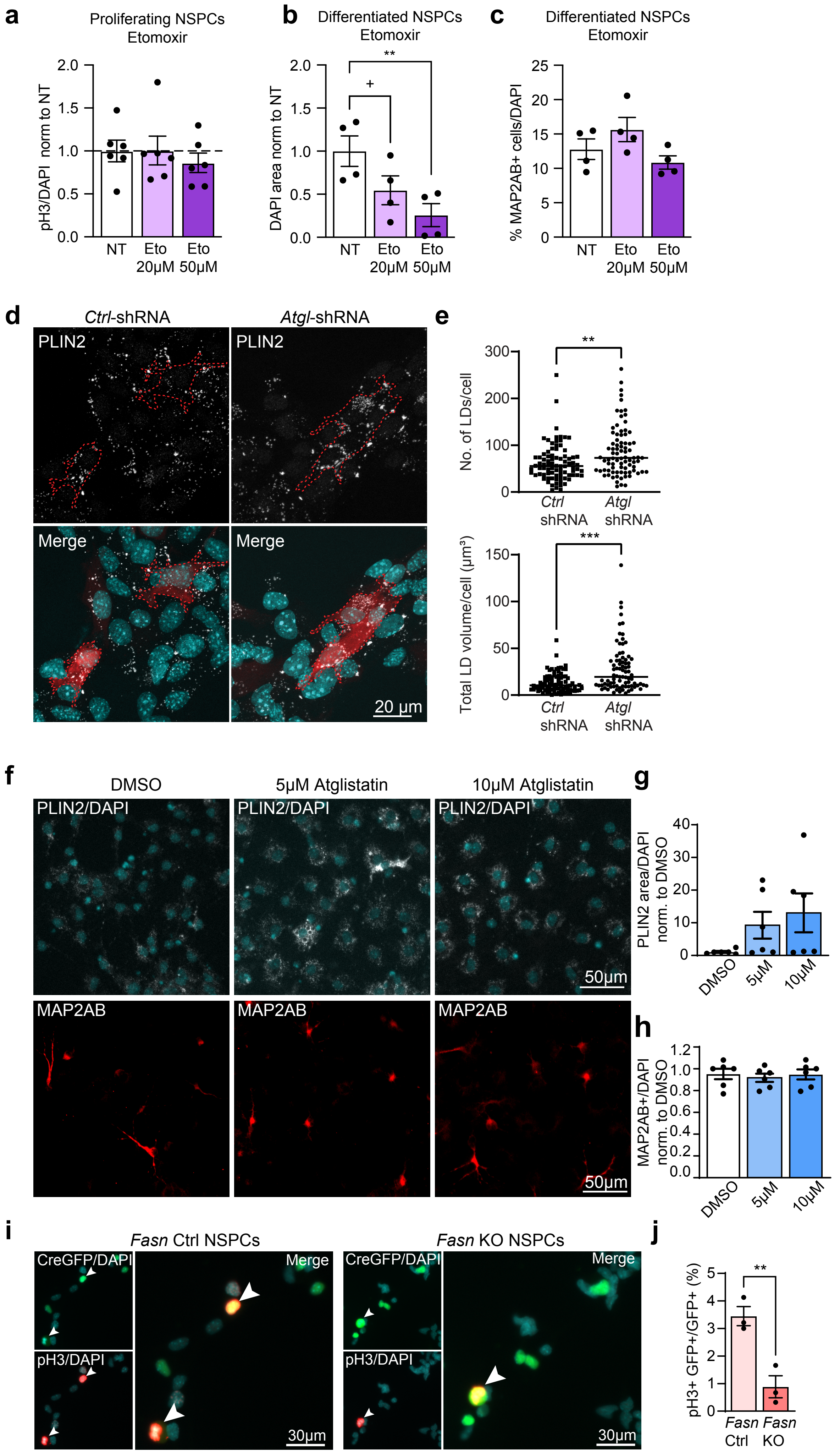

a
Proliferating NSPCs
Etomoxir
pH3/DAPI norm to NT
2.0
1.5
1.0
0.5
0.0
NT
Eto 20μM
Eto 50μM
b
Differentiated NSPCs
Etomoxir
DAPI area norm to NT
**
+
NT
Eto 20μM
Eto 50μM
c
Differentiated NSPCs
Etomoxir
% MAP2AB+ cells/DAPI
25
20
15
10
5
0
NT
Eto 20μM
Eto 50μM
d
Ctrl-shRNA
Atgl-shRNA
PLIN2
Merge
20 μm
e
No. of LDs/cell
300
200
100
0
**
Ctrl shRNA
Atgl shRNA
Total LD volume/cell (μm³)
150
100
50
0
***
Ctrl shRNA
Atgl shRNA
f
DMSO
5μM Atglistatin
10μM Atglistatin
PLIN2/DAPI
MAP2AB
50μm
g
PLIN2 area/DAPI norm. to DMSO
40
30
20
10
0
DMSO
5μM
10μM
h
MAP2AB+/DAPI norm. to DMSO
1.2
1.0
0.8
0.6
0.4
0.2
0.0
DMSO
5μM
10μM
i
Fasn Ctrl NSPCs
Fasn KO NSPCs
CreGFP/DAPI
pH3/DAPI
Merge
30μm
j
pH3+ GFP+/GFP+ (%)
5
4
3
2
1
0
**
Fasn Ctrl
Fasn KO

Supplementary Figure 7: Manipulating the build-up or breakdown of LDs decreases NSPC proliferation, related to Figure 7.

a) The ratio of pH3 positive NSPCs over all cells does not change with Etomoxir under proliferation conditions (n=6 coverslips shown as dots, from 3 independent experiments, bars indicate the mean value +/-SEM, one-way ANOVA; non-significant). **b)** The number of differentiated NSPCs, treated two days with Etomoxir, are significantly decreased (n=4 coverslips shown as dots, from 2 independent experiments, bars indicate the mean value +/-SEM, one-way ANOVA, Holm-Sidak correction, p-value=0.0764 NT vs 20μM, p-value=0.0191 NT vs 50μM). **c)** Despite decreased cell numbers, the proportion of MAP2AB+ cells does not change with etomoxir (n=4 coverslips shown as dots, bars indicate the mean value +/-SEM, one-way ANOVA, non-significant). **d)** Representative images are maximum intensity projections of *Ctrl*-shRNA and *Atgl*-shRNA transfected proliferating NSPCs (red). PLIN2 is shown in white. DAPI-positive nuclei (blue). **e)** Number of LDs and total volume of LDs significantly accumulate upon *Atgl*-knockdown compared to *Ctrl*-shRNA transfected NSPCs. (n= 83 Atgl-knockdown cells and 84 *Ctrl*-shRNA cells from 4 coverslips from 2 independent experiments, Mann Whitney test, two-tailed, p-value=0.0017 for LDs/cell, p-value<0.0001 for LD volume/cell). **f)** Representative images of differentiated NSPCs treated with Atglistatin the first two days of differentiation. PLIN2 (white), MAP2AB (red), DAPI (blue). **g)** PLIN2 area covered, normalized to cell numbers, shows a mild non-significant increase after Atglistatin treatment during the first two days of differentiation (n=9 coverslips shown as dots, from 3 independent experiments, bars indicate the mean value +/-SEM, one-way ANOVA, non-significant). **h)** The number of MAP2AB+ cells does not change with Atglistatin (n=9 coverslips shown as dots, from 3 independent experiments, bars indicate the mean value +/-SEM, one-way ANOVA, non-significant). **i)** Representative images of *Fasn* Ctrl NSPCs and *Fasn* KO NSPCs, 4 days after infection with a Cre-GFP virus (green). Mitotic cells are shown by pH3 (red, arrowheads). **j)** The number of pH3+ NSPCs among Cre-GFP+ NSPCs significantly decreases in *Fasn* KO NSPCs (n=3 coverslips, from 3 independent experiments, bars indicate the mean value +/-SEM, t-test, two-sided, p-value=0.0084). Asterisks indicate the following p-value: *<0.05. **<0.01. ***<0.001. +=0.0764.

**Antibody**	**Species**	**Dilution**	**Provider**	**Protocol used**
GFP	Chicken	1:500	Abcam (ab13970)	Triton
SOX2	Goat	1:500	RnD systems (AF2018)	Saponin
PLIN2	Rabbit	1:1000	Abcam (ab52356)	Saponin
TUBULIN-α	Rat	1:1000	Bio-Rad (MCA77G)	Saponin
phospho HISTONE H3	Mouse	1:1000	Abcam (ab14955)	Triton
RFP	Rabbit	1:500	Abcam (ab62341)	Triton
MAP2AB	Mouse	1:500	Sigma (M2320)	Triton and Saponin
GFAP	Chicken	1:500	Aves Lab (GFAP)	Triton and Saponin
4-HNE	Mouse	1 :250	RnD systems (Mab3249)	Triton

Table 1: Antibodies used in this study
