# Supplementary material for: Lipid droplet availability affects neural stem/progenitor cell metabolism and proliferation
Source: Nat Commun. 2021 Dec 21;12:7362. doi: 10.1038/s41467-021-27365-7 (PMC8692608; doi:10.1038/s41467-021-27365-7)
Supplement: Supplementary file 2 — Description of Additional Supplementary Files [file 41467_2021_27365_MOESM2_ESM.docx]

**Description of Additional Supplementary Files**

File name: Supplementary Data 1. Code scRNASeq analysis Figure 1.R.

Description: Pipeline for scRNAseq analysis presented in Figure 1, using the Seurat package (3.0) of R. The queried datasets are open access and accession numbers are provided in the methods section.

File name: Supplementary Data 2. Code scRNASeq analysis Figure 5.R.

Description: Pipeline for scRNAseq analysis presented in Figure 5, using the Seurat package (4.0.4) of R. The dataset is open access and the accession number is provided in the methods section.

File name: Supplementary Movie 1

Description: Live imaging of eGFP-PLIN2 SVZ NSPCs during 72h of quiescence induction, imaged every 20min, related to Figure 2.

File name: Supplementary Movie 2

Description: Live imaging of eGFP-PLIN2 SVZ NSPCs, related to Figure 4.
