# Supplementary material for: Lipid droplet availability affects neural stem/progenitor cell metabolism and proliferation
Source: Nat Commun. 2021 Dec 21;12:7362. doi: 10.1038/s41467-021-27365-7 (PMC8692608; doi:10.1038/s41467-021-27365-7)
Supplement: Supplementary file 7 — Reporting Summary [file 41467_2021_27365_MOESM7_ESM.pdf]

nature portfolio

Corresponding author(s): Marlen Knobloch
Last updated by author(s): 1.11.2021

Reporting Summary

Nature Portfolio wishes to improve the reproducibility of the work that we publish. This form provides structure for consistency and transparency in reporting. For further information on Nature Portfolio policies, see our Editorial Policies and the Editorial Policy Checklist.

Statistics

For all statistical analyses, confirm that the following items are present in the figure legend, table legend, main text, or Methods section.

n/a	Confirmed	
☐	☒	The exact sample size (*n*) for each experimental group/condition, given as a discrete number and unit of measurement
☐	☒	A statement on whether measurements were taken from distinct samples or whether the same sample was measured repeatedly
☐	☒	The statistical test(s) used AND whether they are one- or two-sided<br>*Only common tests should be described solely by name; describe more complex techniques in the Methods section.*
☐	☒	A description of all covariates tested
☐	☒	A description of any assumptions or corrections, such as tests of normality and adjustment for multiple comparisons
☐	☒	A full description of the statistical parameters including central tendency (e.g. means) or other basic estimates (e.g. regression coefficient) AND variation (e.g. standard deviation) or associated estimates of uncertainty (e.g. confidence intervals)
☐	☒	For null hypothesis testing, the test statistic (e.g. *F*, *t*, *r*) with confidence intervals, effect sizes, degrees of freedom and *P* value noted<br>*Give P values as exact values whenever suitable.*
☒	☐	For Bayesian analysis, information on the choice of priors and Markov chain Monte Carlo settings
☒	☐	For hierarchical and complex designs, identification of the appropriate level for tests and full reporting of outcomes
☒	☐	Estimates of effect sizes (e.g. Cohen's *d*, Pearson's *r*), indicating how they were calculated

Our web collection on statistics for biologists contains articles on many of the points above.

Software and code

Policy information about availability of computer code

Data collection	Data collection, such as image acquisitions, metabolic measurements etc. were performed with the specific instrument softwares installed on the instruments, as detailed in the methods.
Data analysis	Data analysis was performed with the following softwares:<br>Image analysis: ImageJ (version 1.52, https://imagej.nih.gov/), Fiji (Version 2.0.0-rc-69/1.52p), Imaris (Version 9.8, https://imaris.oxinst.com/). Matlab (Version R2019a, https://mathworks.com)<br>scRNA seq data: Seurat 3.0 in R 3.6.1. and Seurat 4.0.4 in R 3.6.1 (Fig. 5)<br>Flow Cytometry: FlowJo 10.6.2.<br>Details of the software codes used are described in the method section and are available from the corresponding author on reasonable request, as stated under the "Code availability" section in the manuscript

For manuscripts utilizing custom algorithms or software that are central to the research but not yet described in published literature, software must be made available to editors and reviewers. We strongly encourage code deposition in a community repository (e.g. GitHub). See the Nature Portfolio guidelines for submitting code & software for further information.

Data

Policy information about availability of data

All manuscripts must include a data availability statement. This statement should provide the following information, where applicable:

- Accession codes, unique identifiers, or web links for publicly available datasets
- A description of any restrictions on data availability
- For clinical datasets or third party data, please ensure that the statement adheres to our policy

scRNA-seq raw and preprocessed data generated in this study have been deposited in the GEO database under accession code: xxx.
The following previously published datasets analyzed in this study were taken from the GEO database under accession code: GSE109447 [https://www.ncbi.nlm.nih.gov/geo/query/acc.cgi?acc=GSE109447], and GSE106447 [https://www.ncbi.nlm.nih.gov/geo/query/acc.cgi?acc=GSE106447].

Field-specific reporting

Please select the one below that is the best fit for your research. If you are not sure, read the appropriate sections before making your selection.

☒ Life sciences ☐ Behavioural & social sciences ☐ Ecological, evolutionary & environmental sciences

For a reference copy of the document with all sections, see nature.com/documents/nr-reporting-summary-flat.pdf

Life sciences study design

All studies must disclose on these points even when the disclosure is negative.

Sample size	A minimum of n=3 samples was used for each analysis. The nature of the n is described for each experiment in the corresponding figure legends. Sample size determinations are based on previous experience and standards in the field. The low variability between the same type of samples, as indicated by the SEM, confirm that this sampling is sufficient to observe statistically significant differences between treatment conditions. Where variability was expected to be higher (such as for instance in the Seahorse experiments), 4-8 replicates were included per each round of experiment and 5 experiments were performed to validate the observed differences, as indicated in details in the figure legends.
Data exclusions	No data were excluded from the analyses.
Replication	Experiments were repeated as indicated in details for each figure panel and as described in the methods. The replications are shown as individual dots together with the calculated means and variability (standard errors of the means).
Randomization	For Seahorse experiments, cell plating of high-vs low PLIN2-GFP NSPCs on the seahorse plates was randomized for each round of experiments and treatment of wells was performed in a randomized way each round to avoid a plating bias. In all the other cell culture experiments including a treatment, wells were randomly assigned to the different treatment groups.
Blinding	Data acquisition and/or analyses in these studies were performed in a blinded way.

Reporting for specific materials, systems and methods

We require information from authors about some types of materials, experimental systems and methods used in many studies. Here, indicate whether each material, system or method listed is relevant to your study. If you are not sure if a list item applies to your research, read the appropriate section before selecting a response.

Materials & experimental systems

n/a	Involved in the study	
☐	☒	Antibodies
☐	☒	Eukaryotic cell lines
☒	☐	Palaeontology and archaeology
☐	☒	Animals and other organisms
☒	☐	Human research participants
☒	☐	Clinical data
☒	☐	Dual use research of concern

Methods

n/a	Involved in the study	
☒	☐	ChIP-seq
☐	☒	Flow cytometry
☒	☐	MRI-based neuroimaging

Antibodies

Antibodies used	All antibodies used in this study are listed in the method section, Table 1: Antibodies used. Dilutions, provider and ordering numbers

Antibodies used

are given as well as the protocol used.
GFP Chicken 1:500 Abcam (ab13970)
SOX2 Goat 1:500 RnD systems (AF2018)
PLIN2 Rabbit 1:1000 Abcam (ab52356)
TUBULIN-alpha Rat 1:1000 Bio-Rad (MCA77G)
phospho HISTONE H3 Mouse 1:1000 Abcam (ab14955)
RFP Rabbit 1:500 Abcam (ab62341)
MAP2AB Mouse 1:500 Sigma (M2320)
GFAP Chicken 1:500 Aves Lab (GFAP)
4-HNE Mouse 1 :250 RnD systems (Mab3249)

Validation

All antibodies used in this study are from commercial suppliers that have verified the specificity of the antibodies using recombinant proteins or knock-out lines. All the antibodies have been previously used by various laboratories. All secondary antibodies are verified to not give a specific staining without the primary antibody.
Detailed validation information as obtained from the provider's datasheets:
Anti-GFP antibody (ab13970)
- image 5/8: ICC staining GFP in GFP-transfected NIH/3T3 (Mouse embryo fibroblast cell line) cells.
- image 4/8: Each lot is tested using IHC with paraffin-embedded sections using transgenic adult mouse brain with the GFP gene driven by the actin promoter. This results in moderate levels of expression of GFP immunoreactivity in specific cells within cerebral cortex. Specificity: This GFP antibody recognizes all of the GFP derivatives that are derived from the jellyfish, A. victoria. This includes eGFP, YFP, CFP, BFP.

Anti-ADFP antibody (ab52356)
- image 1: WB mouse liver lysate
- image 2: IHC-P mouse liver tissue
- image 3: ICC in HeLa cells

Anti-Histone H3 (phospho S10) antibody [mAbcam 14955] (ab14955)
- image 1: IHC-P human kidney
- image 2, 8: WB HeLa histone preparation +/- colcemid treatment for phospho S10 induction, both also with blocking with histone H3 unmodified peptide (signal), phospho S10 peptide (reduced signal), trimethyl K9 + phospho S10 peptide (no signal), phospho S28 peptide (signal)
- image 7: WB HeLa lysate control, calyculin A treated, calyculin A + alkaline phosphatase treated
collaborator testing:
- image 3, 5: ICC in mouse 10T1/2 cells, Indian muntjac cells
- image 4: flow cytometry in HeLa cells

Anti-RFP antibody (ab62341)
- image 1: WB of different amounts of recombinant RFP

SOX2 Goat RnD systems (AF2018):
Antibody already used for Western blot on lysates of D3 mouse embryonic stem cell line, NTera-2 human testicular embryonic carcinoma cell line, F9 mouse teratocarcinoma stem cells, and rat cortical stem cells and for detection of SOX2-regulated genes by Chromatin Immunoprecipitation of BG01V human embryonic stem cells. It was also used for Immunocytochemistry on mouse and rat cortical stem cells as well as on ADLF1 and FAB2 Stem Cell Lines.

TUBULIN-alpha Rat Bio-Rad (MCA77G)
Antibody used for the detection of cytoskeletal elements in Drosophila embryos, in the ctenophore Pleurobrachia pileus, in the cnidarian Clytia hemisphaerica, in T. brucei, Arabidopsis thaliana pollen and in rat neurons by immunofluorescence. It was also used for protein loading control of several lysate coming from various cell types such HeLa and C6 rat glioma and species such as human, mouse, yeast and Drosophila lysates.

MAP2AB Mouse Sigma (M2320)
specificity of this antibody was established by immunoblot. This antibody reacts with human, bovine, rat, mouse, Xenopus, salamander, and quail tissue or cells by immunocytochemical techniques utilizing either a fluorescent or peroxidase label, or by immunohistochemistry

GFAP Chicken Aves Lab (GFAP)
Antibody already used for detection of intermediate filament protein by immunohistochemistry and immunofluorescence on mixed cortical mouse brain cultures as well as on mouse brain sections.

4-HNE Mouse RnD systems (Mab3249)
Antibody already used for Western blot and Simple Western on HepG2 human hepatocellular carcinoma cell line. It was also used for detection of oxidative damage on human prostate sections and human prostate cancer tissues by immunohistochemistry.

Eukaryotic cell lines

Policy information about cell lines

Cell line source(s)

All NSPCs used in this study were derived from adult mice by ourselves. Cells were kept as "lines" for many passages (up to P25).

Authentication

None of the cells used were authenticated

Mycoplasma contamination	All NSPCs used in this study were regularly tested for mycoplasma contamination and were negative.
Commonly misidentified lines (See ICLAC register)	No commonly misidentified lines were used in this study.

Animals and other organisms

Policy information about studies involving animals; ARRIVE guidelines recommended for reporting animal research

Laboratory animals	NSPCs used in this study were isolated from 6-8-week-old C57/Bl6 male mice, provided by Janvier, France. Fucci NSPCs were isolated from FUCCI-Mice (B6.Cg-Tg(FUCCIS/G2/M)#504Bsi Tg(FUCCIG1)#596Bsi), purchased through RIKEN (Japan). Housing conditions were as following: dark/light cycle 12/12, ambient temperature around 21-22°C and humidity between 40 and 70% (55% in average).
Wild animals	The study did not involve wild animals.
Field-collected samples	The study did not involve field-collected samples
Ethics oversight	All experiments including animals were carried out in compliance with the Swiss law after approval from the local authorities (Cantonal veterinary office, Canton de Vaud, Switzerland).

Note that full information on the approval of the study protocol must also be provided in the manuscript.

Flow Cytometry

Plots

Confirm that:

☒ The axis labels state the marker and fluorochrome used (e.g. CD4-FITC).

☒ The axis scales are clearly visible. Include numbers along axes only for bottom left plot of group (a 'group' is an analysis of identical markers).

☒ All plots are contour plots with outliers or pseudocolor plots.

☒ A numerical value for number of cells or percentage (with statistics) is provided.

Methodology

Sample preparation	NSPCs were prepared for Flow Cytometry as also specified in details in the methods. NSPCs were trypsinized and if applicable incubated for 10min at 37°C with the cell viability dye Calcein Red (C34851 ThermoFisher, 1:1000 in 1x PBS). Cells were washed twice with PBS and kept in EDTA-DPBS (E8008, Merck Millipore) at 4°C during the sorting process.
Instrument	MoFlo Astrios EQ cell sorter (Beckman Coulter, UNIL FACS facility), Cytoflex S Flow cytometer (Beckman Coulter)
Software	Flow Cytometry data were acquired with the instrument software and analyzed with FlowJo 10.6.2.
Cell population abundance	low and high PLIN2-GFP NSPCs were sorted in purity mode as 25% lowest and 25% highest of the total PLIN2-GFP population.
Gating strategy	Gating strategies are shown for each experiment in the supplementary information. They were established using single color stains where applicable. In general, the first gating is done on viable cells to exclude debris and dead cells, followed by the selection of single cells (excluding doublets or clumps). Only then was the specific fluorophore (such as for instance GFP) taken into account.

☒ Tick this box to confirm that a figure exemplifying the gating strategy is provided in the Supplementary Information.
